# Trends in Photopolymerization 3D Printing for Advanced Drug Delivery Applications

**DOI:** 10.1021/acs.biomac.4c01004

**Published:** 2024-12-03

**Authors:** Yu Hu, Zhi Luo, Yinyin Bao

**Affiliations:** † Department of Biomedical Engineering, Southern University of Science and Technology, Shenzhen 518055, Guangdong, P.R. China; ‡ Department of Chemistry and Applied Biosciences, ETH Zurich, Vladimir-Prelog-Weg 1, 8093 Zurich, Switzerland; § Department of Chemistry, Faculty of Science, University of Helsinki, 00014 Helsinki, Finland

## Abstract

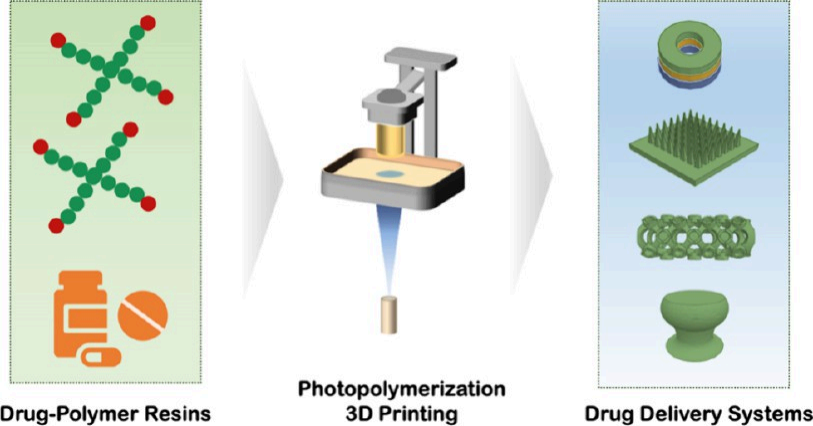

Since its invention in the 1980s, photopolymerization-based 3D printing has attracted significant attention for its capability to fabricate complex microstructures with high precision, by leveraging light patterning to initiate polymerization and cross-linking in liquid resin materials. Such precision makes it particularly suitable for biomedical applications, in particular, advanced and customized drug delivery systems. This review summarizes the latest advancements in photopolymerization 3D printing technology and the development of biocompatible and/or biodegradable materials that have been used or shown potential in the field of drug delivery. The drug loading methods and release characteristics of the 3D printing drug delivery systems are summarized. Importantly, recent trends in the drug delivery applications based on photopolymerization 3D printing, including oral formulations, microneedles, implantable devices, microrobots and recently emerging systems, are analyzed. In the end, the challenges and opportunities in photopolymerization 3D printing for customized drug delivery are discussed.

## 1 Introduction

Drug delivery systems play a crucial role in ensuring the safe and efficient administration of therapeutic agents.^1,2^ However, traditional delivery techniques face numerous challenges, such as limited control over drug release, difficulties in accommodating patient-specific variations, and suboptimal utilization of active pharmaceutical ingredient.^3,4^ Recent advances in medical devices pose further challenges in the drug incorporation and release strategies that need to meet the requirements of both drug delivery and medical manufacturing.^1,6^ Additionally, concerns regarding side effects and drug wastage necessitate more innovative formulation technologies.^7^

Originated in the late 1980s from stereolithography (SLA) and initially applied in the industrial fields, 3D printing has shown significant potential in revolutionizing drug delivery system.^8,9^ In 2015, the Food and Drug Administration (FDA) approved the first 3D-printed drug, Spritam (levetiracetam), for the treatment of epilepsy.^10,11^ Utilizing powder bed binding technology, this approval marked the recognition of 3D printing technology in pharmaceutical manufacturing by regulatory authorities, sparking a new wave of industry innovation and development.^12^ 3D printing offers drug delivery systems unique advantages, such as tunable control over drug release kinetics, customized local treatment and the ability to prepare multifunctional drug carriers.^9^ By integrating medical imaging data with CAD software, personalized drug delivery systems can be precisely designed, such as drug-eluting devices and implants with customized geometries, and drug formulations.^13^ Furthermore, the combination of various materials, including polymers and nanomaterials, through composite printing adds multiple functionalities to drug delivery systems (e.g., shape-memory and stimuli-responsiveness), catering to diverse treatment needs.^14,15^ The rapid evolution of 3D printing technologies also accelerates the development and production of drug formulations such as multidrug tablets and smart capsules, enhancing personalization according to individual requirement.^16^

The 3D printed drugs market was valued at USD 638.6 million in 2019 and is projected to grow at a compound annual growth rate of 15.2%, reaching USD 2064.8 million by 2027.^17^ 3D printing technologies adopted in pharmaceutical formulation includes several different methods, such as fused deposition modeling (FDM), selective laser sintering (SLS), and inkjet printing.^18^ Among these technologies, FDM is the most widely used, utilizing polymer filaments that are heated and extruded through a nozzle to form 3D shapes in a layer-by-layer manner.^19^ Despite its slow printing speed and rough surface finish, FDM remains extensively adopted.^20^ On the other hand, SLS technology employs laser sintering for solidification, requiring materials with high heat stability, thus limiting the range of drugs and excipients.^21^ Inkjet printing, though with lower precision, is commonly used for tissue engineering and drug-loaded microneedles.^22^ Overall, each technique possesses unique characteristics and applications, offering abundant possibilities for innovation in the field of pharmaceutical formulations.^23^

Photopolymerization-based 3D printing (typically vat photopolymerization) has attracted significant attention in pharmaceutical field, due to the advancements in photochemistry, polymer science and optical technology.^24,25^ This technique utilizes light radiation to initiate polymerization and/or cross-linking reactions in liquid resin materials, forming 3D structures.^26^ The high precision of light control and material flexibility of photopolymerization-based 3D printing enables the accurate fabrication of complex microstructures for drug carriers and medical devices.^27,28^ Specifically, photopolymerization-based 3D printing enables the production of sophisticated delivery systems such as implantable devices,^29,30^ microneedle patches,^31,32^ and drug-eluting stents.^33,34^ These advancements provide novel treatment solutions for conditions such as diabetes,^31^ tumors,^32^ and pain management.^35^ Recently, novel drug delivery applications have been explored, ranging from transdermal patches^36^ and suction cups,^37^ to detoxification devices^38^ and remote-controlled microrobots.^39,40^

**Figure 1:**
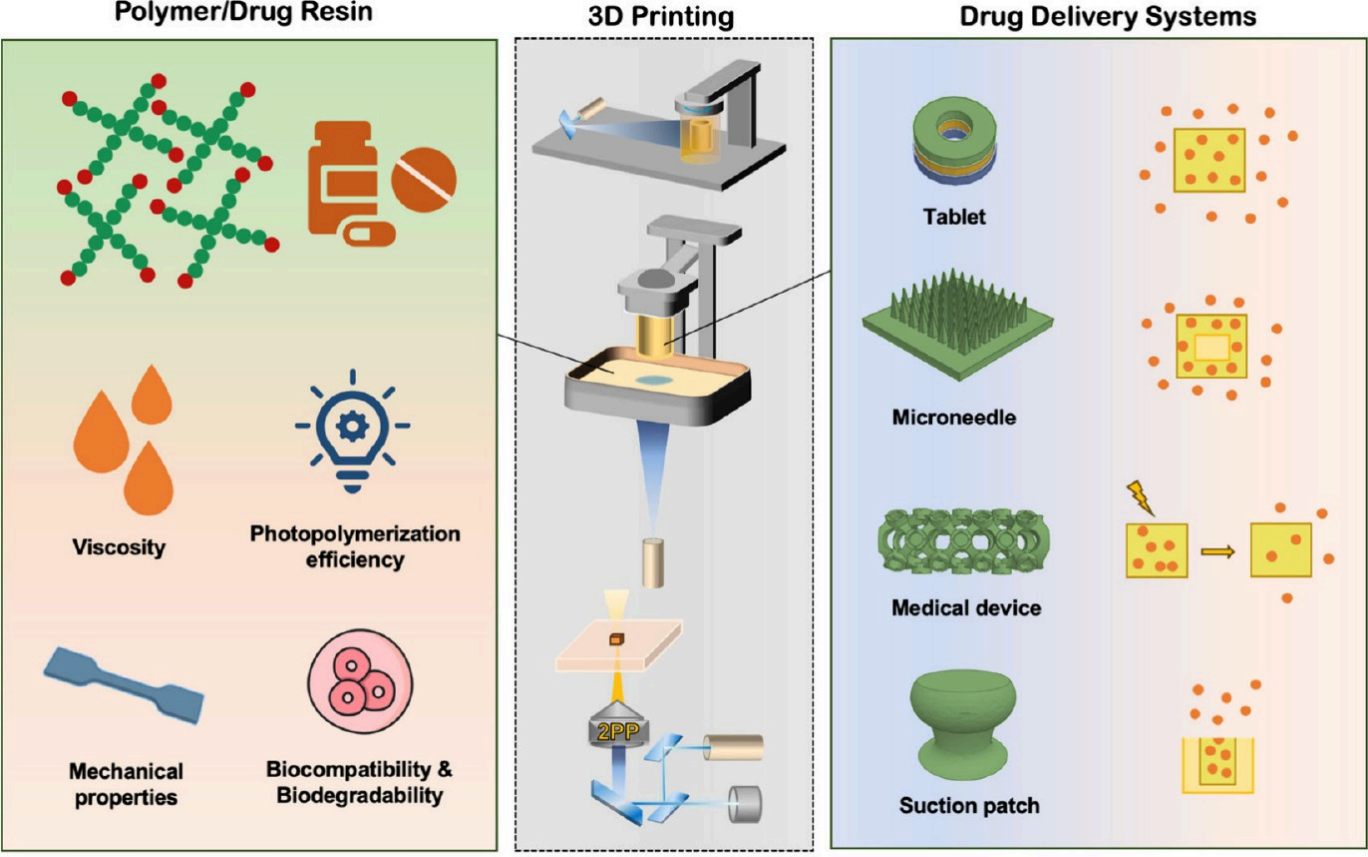
Schematic diagram of photopolymerization 3D printing for drug delivery.

The chemical versatility of photopolymerization materials, especially the photopolymers with controlled biodegradability and tunable functions (e.g., shape-memory), further expands the capabilities of personalized drug delivery systems.^41,42^ While existing reviews have primarily focused on broader aspects of 3D printing technologies for biomedical applications, this review aims to provide a critical analysis of recent trends (especially in last three years) in advanced drug delivery systems by photopolymerization 3D printing, which represent a rapidly evolving part within the field. We first provide an overview of representative and emerging photopolymerization techniques, and the corresponding biocompatible and biodegradable photopolymers. Then we illustrate the associated drug loading/release mechanisms in photopolymerization 3D printing systems, which are often overlooked in most existing reviews. Next, we evaluate the new trends in the application of vat photopolymerization for advanced drug delivery, with a focus on novel drug formulations (e.g., by volumetric printing), microneedles (e.g., for vaccine and protein delivery), drug-eluting devices and implants (e.g., shape memory stents), and emerging 3D printing systems (e.g., microrobots and suction patch) (Figure 1). To be more focused, we emphasize the applications of 3D printing systems only related to release, delivery or removal of drug molecules, and will thus not discuss the studies merely on tissue engineering or medical device fabrication.

## 2 Photopolymerization-Based 3D Printing Methods

In this section, we summarize the representative 3D printing methods based on vat photopolymerization, elucidating their working principles. Since a number of reviews covering 3D printing techniques have been published,^26,27,43^ we focus on the most commonly used and also recently emerging photopolymerization methods, including conventional photopolymerization techniques, nonlinear photopolymerization, volumetric printing, and their derived methods. The emerging techniques such as xolography and upconversion photopolymerization, are also briefly discussed, although they have not been used for drug delivery. Typically, the photopolymerizable resin formulations used in most of these printing methods include photoinitiators (PIs), monomers or photopolymers, solvents/diluents, photoabsorbers or dyes, and radical inhibitors.^44,45^

### 2.1 Conventional 3D Printing Based on Linear Photopolymerization

**Figure 2:**
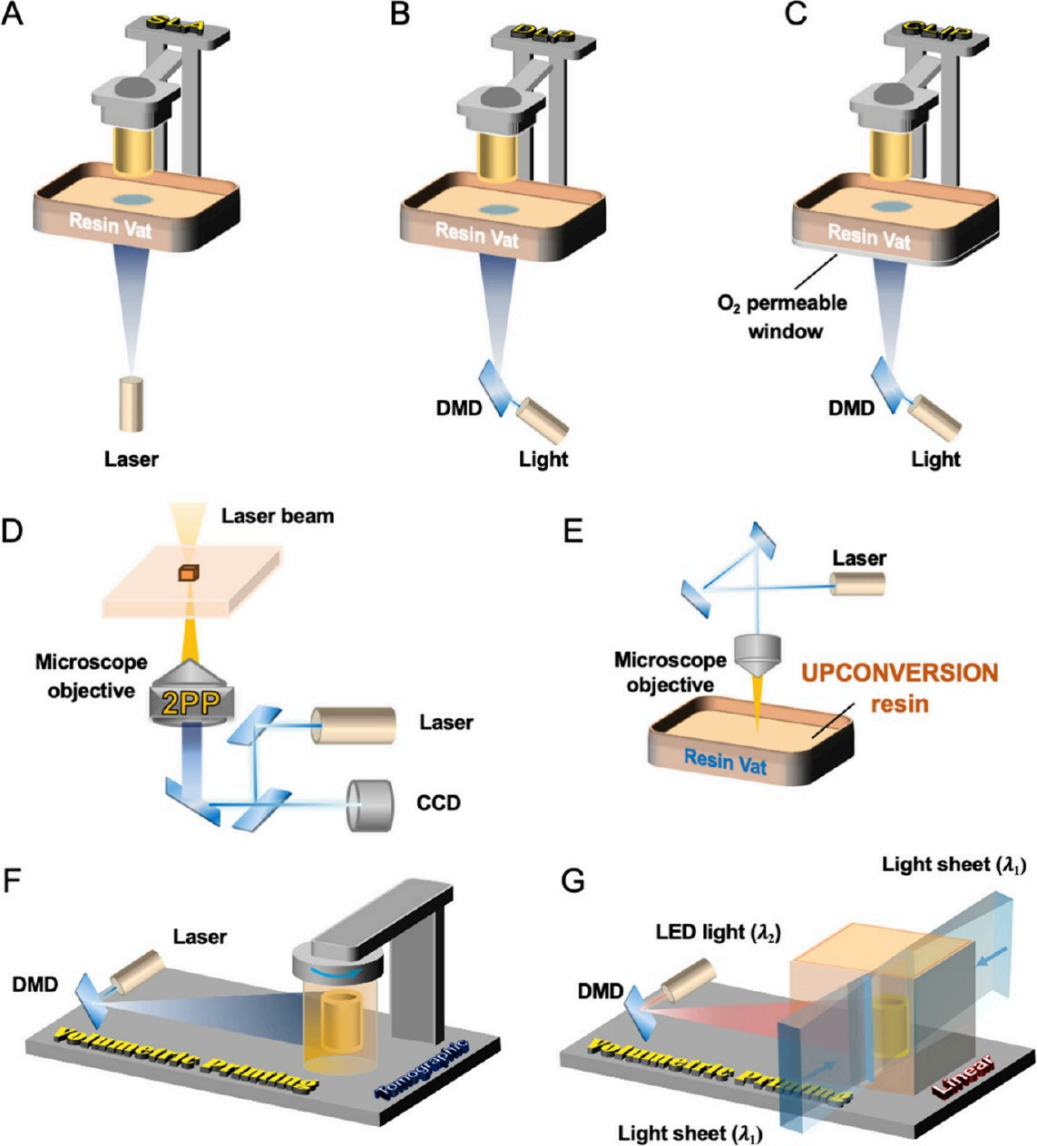
Schematic illustration of representative vat photopolymerization techniques. (A) SLA, (B) DLP, (C) CLIP, (D) 2PP, (E) upconversion printing, (F) tomographic volumetric printing and (G) xolography. Partially adapted with permission under a Creative Commons CC-BY 4.0 from ref (28).

Conventional photopolymerization 3D printing is usually based on one-photon absorption, and thus the resin absorption is linearly correlated with the light intensity.^46^ These photopolymerization techniques involve layer-by-layer fabrication of 3D objects by polymerization/cross-linking of monomers or prepolymers using photoinitiators under exposure to a light source.^47^ Depending on the type of light source and the specific working mechanism, linear photopolymerization 3D printing technologies are represented by stereolithography (SLA, Figure 2A), digital light processing (DLP, Figure 2B), and continuous liquid interface production (CLIP, Figure 2C).

SLA is a pioneering technology in 3D printing, initially patented in the 1980s by French engineers Olivier de Witte, Jean-Claude André, and Alain Le Méhauté,^48^ although their project was abandoned for business reason. Charles Hull independently filed a patent in 1986, corning the term SLA.^43,49^ This technology is widely used in medicine,^50^ dentistry,^51^ and automotive industries.^52^ Its core principle involves using a laser to selectively solidify layers of liquid photosensitive monomers/polymers, constructing objects through layer-by-layer solidification.^53^ Currently, SLA is characterized by high maturity, stable printing processes, and a variety of commercial suppliers.^54^ Unlike FDM, SLA exhibits particularly low anisotropy in strength due to the uniformity of the solidified layers.^55^ Despite its precision and capability to print structurally complex and finely detailed objects, SLA faces certain limitations for biomedical applications. The range of biocompatible resins available for cationic or radical photopolymerization is limited.^56^ High viscosity of the resin can cause difficulties in achieving precise printing details.^57^ Preheating the resin can reduce its viscosity, making it easier to work with and improving printing accuracy.^58,59^ The SLA process also typically involves postprocessing steps such as cleaning, support removal, polishing, and additional curing.^60^ However, the limited variety of photosensitive resin materials and the potential health risks associated with PI restrict the widespread use of SLA technology in the pharmaceutical field.^43^

In recent years, the development of microstereolithography (micro-SLA) technology has achieved precision levels down to a few micrometers or even smaller.^61^ This advancement relies on several key factors: advanced optical techniques, precise optical system design, high-resolution beam control, and optimized selection of photosensitive resins.^62^ Compared to traditional SLA technology, micro-SLA is more focused on the manufacturing of microcomponents and microstructures, capable of achieving fine features at the micronanoscale level and suitable for applications requiring extremely high spatial accuracy.^63^

Polymer jetting (PolyJet) technology, also known as multijetting (MultiJet) technology, was pioneered by Objet Geometries in 2000.^64,65^ Unlike stereolithography, printing materials are jetted through print nozzles in the form of droplets or liquid jets.^66^ The droplets are deposited and rapidly solidified through auxiliary curing mechanisms, typically using ultraviolet light, to build each layer of patterned structures and provide high printing resolution.^67^ Multijet devices easily control material composition, enabling strong multimaterial capabilities, adept at constructing support structures or multimaterial structures with different properties and colors without additional assembly steps.^68^ Furthermore, it requires simple postprocessing.^69^ These are the primary advantages of material jetting. On the other hand, material jetting’s printing speed is similar to SLA, suitable for 3D printing in fields such as organ models and jewelry.^70,71^ Companies like Stratasys and 3D Systems offer PolyJet printers, such as the Stratasys J series and the ProJet CJP 660Pro, which support various engineering and biomedical-grade materials. In the medical field, hospitals use PolyJet printers to create realistic and transparent patient kidney models for surgical planning, assisting in complex procedures.^71^ However, PolyJet printers and materials are expensive, and the printing speed is relatively slow, limiting large-scale production.^72^

As the second generation of SLA 3D printing, DLP was proposed by Larry Hornbeck of Texas Instruments in 1987.^73,74^ The core component of DLP is the digital micromirror device (DMD), an optical microstructure composed of millions of tiny movable mirrors arranged on a chip.^75^ Each micromirror represents a pixel in the image, with the resolution depending on the number of mirrors. These micromirrors control light reflection by switching between two positions.^76^ In a DLP system, the DMD chip is placed between the light source and the projection surface.^77^ Under irradiation, light is reflected and then focused through a projection lens onto the surface to create an image.^78^ Unlike SLA, DLP involves layer-by-layer curing of photosensitive resin using the DMD-based projector, enabling rapid prototyping.^79^ Each layer’s image generates a light-induced polymerization reaction, solidifying the resin to form a thin layer of the part.^79^ The printing platform then moves one layer, and the projector process the next layer in a cyclical manner until printing is complete, resulting in high accuracy and fast speed.^80^ Similar to SLA, high resin viscosity can hinder printing accuracy and cause problems with resin flow.^81^ To address this, one can optimize resin formulations and preheat the resin to achieve lower viscosities suitable for DLP printing without compromising print quality.^77^ However, to ensure high precision (below 50 μm), the projection size is generally small, and the lifespan of the light source and projection equipment is limited.^80^ DLP printing technology has been widely applied in research to manufacture customized drug delivery systems, such as oral medications,^82^ and transdermal patches.^83^

As the demand for faster printing speeds continues to rise, researchers have begun exploring more efficient and flexible methods. CLIP, invented by the group of DeSimone in 2015 and commercialized by Carbon, is an advanced printing technology that enables continuous photopolymerization in 3D.^84^ This technique employs a transparent, oxygen-permeable Teflon film as the bottom of the resin vat, allowing light and oxygen to pass through in high rate. The oxygen inhibition effect creates a “dead zone” at the resin vat’s bottom, preventing photopolymerization in this thin region and enabling continuous printing. In CLIP, UV light cures the resin above the “dead zone”, maintaining a stable liquid interface to ensuring continuous curing. This process transforms traditional 3D printing into an adjustable photochemical process, eliminating the layered steps typically involved. Compared to DLP, CLIP technology improves printing speed (∼0.5 cm/min) by up to 100 times, achieving extremely fast manufacturing, high surface smoothness, and complete isotropy in produced parts.

Despite its advantages, CLIP technology also faces challenges similar to SLA and DLP, particularly with high-viscosity resins. To address this, Lipkowitz and colleagues developed the ‘injection continuous liquid interface production (iCLIP)’ method.^85^ This method repurposes the oxygen-filled “dead zone” by injecting additional materials, which is mechanically fed with resin at elevated pressures through microfluidic channels dynamically created and integral to the growing part. This method significantly accelerates printing speeds, accommodates higher-viscosity resins, and allows for the simultaneous use of multiple resins, enabling the rapid production of complex, multimaterial structures.

Additionally, roll-to-roll continuous liquid interface production (r2rCLIP) combines CLIP technology with roll-to-roll manufacturing, facilitating the efficient production of large-scale particles.^86^ This technology is suitable for preparing ceramic and hydrogel particles. The Mirkin group developed a high area rapid printing (HARP) method, which is a dead-zone-free rapid SLA printing technique.^87^ It achieves continuous printing of large areas and rapid vertical printing speed by floating UV-curable resin on a flowing immiscible fluorinated oil bed. Conventional SLA 3D printing typically achieves printing speeds of around 10–20 mm per hour and a resolution of 50–100 μm. This method largely improves SLA printing speed, reaching a continuous vertical printing rate of over 430 mm per hour and a volumetric throughput of 100 L per hour. Overall, due to its high precision and speed, CLIP technology has been utilized to fabricate various drug delivery devices, in particular customized microneedles for drug and vaccine delivery, enabling tailored therapeutic approaches for diverse medical applications.^88−90^

### 2.2 Nonlinear Photopolymerization 3D Printing

Although SLA, DLP, and CLIP technologies have greatly advanced the field of 3D printing, their inherent limitations prevent achieving resolutions beyond the micrometer scale. In contrast, nonlinear photopolymerization 3D printing, such as two-photon polymerization (2PP, Figure 2D), leverages the unique properties of photon absorption to achieve nanometer-level precision. This fundamentally expands the possibilities of additive manufacturing.

Two-photon 3D printing, also known as direct laser writing (DLW) technology, is an advanced additive manufacturing technique.^91^ This technology utilizes a laser light source to construct three-dimensional objects with extreme precision. Photopolymerization only occurs at the focal point where two photons are simultaneously absorbed, enabling resolutions of 100–300 nm. This high precision makes 2PP suitable for manufacturing complex and finely structured components.^92^

Despite its advantages, two-photon 3D printers are generally much more expensive than other photopolymerization printers. In addition, they have relatively slower printing speeds due to the scan mode of point light resource.^93^ Nevertheless, its outstanding resolution and ability to fabricate complex structures makes 2PP highly valued in the production of miniature devices for biomedical applications.^94^ For example, Mandt et al. used a gelatin-based material to construct a microfluidic model mimicking placental transport.^95^ This model can investigate the placental microenvironment under various pharmacological, clinical, and biological scenarios, exploring its effects on development and transport processes, particularly were altered nutrient transport poses health risks for the fetus. Additionally, 2PP is used to manufacture complex and detailed microneedle array master molds for the production of dissolvable and hydrogel-based microneedle arrays for drug delivery.^96,97^ Faraji et al. utilized 2PP to create microneedle with a resolution of 500 nm.^98^ These microneedles, featuring side channels for drug delivery, successful penetrated pig cadavers’ skin, demonstrating the practical applications of 2PP in creating advanced drug delivery devices.

To lower the cost and speed up the printing, another nonlinear photopolymerization strategy, upconversion photopolymerization 3D printing, emerged recently. This technique uses upconversion materials that absorbs near-infrared light and emits visible or UV light, activating the conventional PIs in photosensitive resin (Figure 2E).^99,100^ This method allows precise control over the curing process, enabling the fabrication of complex 3D structures.^46^ Compared to traditional UV or visible light polymerization, upconversion photopolymerization offers advantages such as greater penetration depth, lower phototoxicity, and potentially higher resolution printing.^46,101^ Researchers are actively exploring upconversion materials to expand the wavelength range and improve transparency and multifunctionality.^102,103^ Rocheva et al. demonstrated the feasibility of near-infrared light (NIR)-induced polymerization with upconversion nanomaterials for rapid 3D prototyping.^104^ Using triplet fusion upconversion capsules, Congreve and co-workers achieved volume printing under less than 4 mW of continuous-wave excitation, reaching a printing accuracy of 50 μm.^99^ While this technology is promising, challenges such as existing metal species, material biocompatibility and high nanoparticle concentration need to be addressed before drug delivery applications.

### 2.3 Volumetric 3D Printing

Currently, most 3D printing methods, including FDM, SLA, and DLP, rely on layer-by-layer construction. This approach often requires support materials for printing hollow or overhanging structures, limiting the precise manufacturing of complex geometries and extending the durations needed for large structures. Enhancing printing efficiency and accuracy remains a critical challenge in 3D printing research.

The concept of volumetric 3D printing, first proposed by Shusteff and co-workers in 2017, introduces a novel method fundamentally differs from traditional layer-by-layer paradigm.^105^ This method superimposes patterned light fields from multiple beams within a photosensitive resin, enabling the formation of complex, nonperiodic three-dimensional volumes without the need for substrates or support structures, significantly accelerating the printing process. In 2019, Kelly et al. introduced the computational axial lithography (CAL, Figure 2F), a technique that decomposes a three-dimensional object into two-dimensional images and projects light from different angles to solidify photosensitive liquid into the desired shape within 30–120 s, achieving precision up to 300 μm.^106^ In parallel, Christophe’s team also advanced tomographic volumetric printing, achieving high resolution (80 μm) by a rotating cylindrical resin container and light from a DLP modulator, enabling high-speed printing.^107^ Riccardo and co-workers applied this technology to bioprinting, manufacturing cell-loaded structures of varied sizes and shapes within seconds to tens of seconds, allowing rapid formation of biologically inked centimeter-scale fine structures.^108^ Rodríguez-Pombo and colleagues recently applied volumetric printing to manufacture oral pharmaceutical dosage forms, specifically designing and simultaneously printing torus and cylinder shapes.^109^ They optimized critical printing parameters for six formulations containing paracetamol.

While tomographic volumetric printing projects the entire shape of an object from multiple angles for solidification, new linear volumetric printing technologies emerged utilizing a thin light sheet beam for image projection and curing. Stefan Hecht and colleagues developed an innovative linear volumetric 3D printing method,^110^ introducing dual-color technique with photoswitchable PI to induce local polymerization within a confined monomer volume, and named it xolography (Figure 2G). The mechanism behind xolography employs a two-color, two-step photoinitiation process. Photoswitchable molecule absorbs light at wavelength λ1, entering an idle intermediate electronic state. If pre-excited molecules absorb light at wavelength λ2, they are further excited to a higher energy state, initiating polymerization in the presence of an electron donor (typically amines). These advancements promise enhanced efficiency, accuracy, and the capability to fabricate complex structures rapidly. Xolography offers significantly improved resolution (approximately 25 μm in the x and y directions) and volume generation rates (55 mm^3^ s^–1^), potentially revolutionizing rapid production across micro to macroscopic scales. Furthermore, Hahn et al. achieved a peak printing rate of 7 × 10^6^ voxels s^–1^ with a voxel volume of 0.55 μm^3^ using two -color two-step absorption.^111^ Xolography has been also made continuous by integrating a flow resin cell, which potentially enables the up-scaling of the production.^112^ In addition, xolography 3D printing of polymeric multimaterials has also been achieved, with the assistance of reversible addition–fragmentation chain transfer (RAFT) polymerization process.^113^

## 3 Photopolymers for 3D Printing of Drug Delivery Systems

Biocompatible and biodegradable polymers have shown tremendous potential in drug delivery systems, such as nanomedicine and immunotherapeutic agents.^114,115^ Biocompatible polymers with 3D printability in particular biodegradable photopolymers represent innovative materials with broad medical applications.^116^ Manufactured through photopolymerization techniques, these materials can form products that gradually degrade within the body, demonstrating unique potential in biomedicine. In recent years, biodegradable 3D printing photopolymers have received increasing attention in the development of customized drug delivery systems.^117,118^ Their controllable degradability allows for the manufacture of miniature drug carriers capable of releasing drugs within the body, achieving sustained and targeted therapeutic effects.^119,120^ The customized drug delivery system empowered by 3D printing enhances treatment efficacy, reduces medication side effects, and supports personalized medicine.^121^

**Figure 3:**
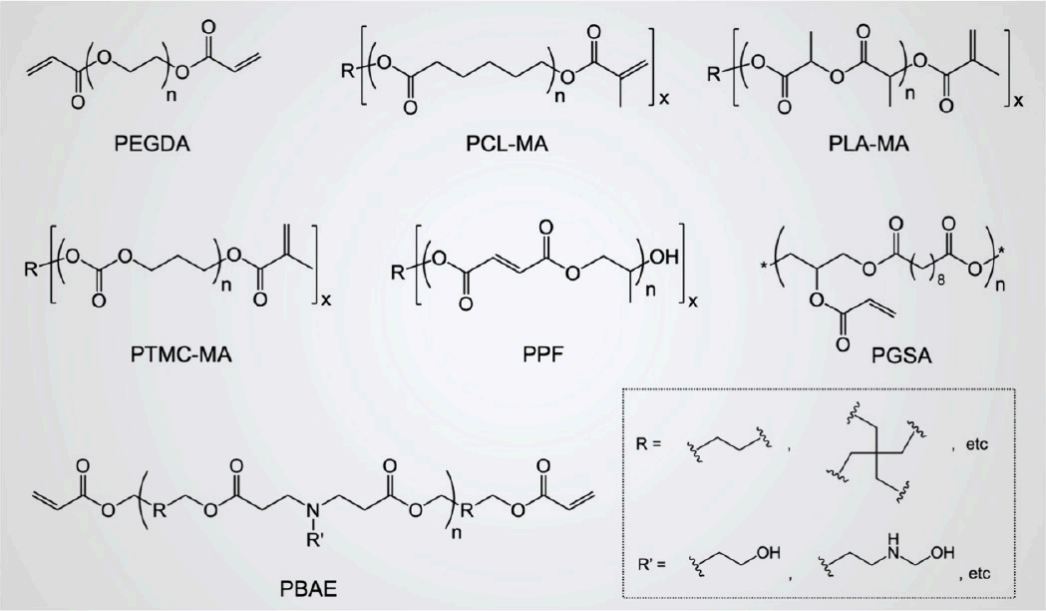
Chemical structures of representative 3D printable photopolymers (x > 1).

Furthermore, biodegradable 3D printing photopolymers have been widely explored in fabricating bioresorbable scaffolds,^122^ and medical devices.^123^ In medical stent and soft tissue engineering, these materials can be fabricated into various shapes and structures to support tissue growth and repair.^124,125^ Over time, these implants and scaffolds degrade, providing temporary support to newly formed tissue, promoting healing, and ultimately being metabolized by the body, thereby reducing the need for secondary surgeries.^119,123,126^ This section introduces representative 3D printing photopolymers including both biodegradable photopolymers and nondegradable but commonly used photopolymers for biomedical applications (Figure 3). Their physicochemical properties and the latest developments are emphasized.

### 3.1 Nonbiodegradable Photopolymers

#### 3.1.1 Polyethylene Glycol Di(meth)acrylate

As a classic biocompatible polymer, polyethylene glycol (PEG) has been widely used as a versatile carrier material in drug delivery.^127^ The broad applications of PEG include pharmaceutical formulation, surface modification, drug conjugation, and nanomedicine.^128,129^ PEG’s excellent biocompatibility and stability make it indispensable in drug delivery system design, enhancing therapeutic effects and minimizing side effects.^130,131^ Polyethylene glycol diacrylate (PEGDA) derived from PEG and acrylic acid (AA), is a commonly used photopolymer in photopolymerization 3D printing.^132^ PEGDA exhibits good cytocompatibility, and fast photopolymerization speed, making it ideal for fabricating biocompatible scaffolds and devices.^133^ This reduces stimulation to surrounding tissues and facilitates the customized design of biomaterials.^134,135^

Combining drugs with PEGDA and utilizing photopolymerization technology enables the fabrication of microcarriers with controllable drug release capability. For instance, in the presence of diphenyl(2,4,6-trimethylbenzoyl) phosphine oxide (TPO) as a PI, PEGDA was SLA printed into multilayered, multidrug pellets with adjustable release rates by incorporating shaping agents.^136,137^ Additionally, DLP can produce PEGDA scaffolds with loaded acetylsalicylic acid (ASA) for drug gradual release.^138^ Studies have indicated that higher levels of PEGDA in printed tablets result in lower drug dissolution rates,^56^ necessitating the addition of shaping agents and other monomers to modulate drug release performance and material degradation.

Using SLA, PEGDA can produce hydrogels loaded with pharmacologically nontoxic photosensitive riboflavin for controlled drug release.^140^ 2PP can fabricate biodegradable, superparamagnetic polymer composites for targeted drug delivery, such as spiral microrobots^141^ composed of magnetite (Fe_3_O_4_) nanoparticles, PEGDA and pentaerythritol triacrylate (PETA). Recently, it was shown that PEG photopolymers can be printed into tough hydrogels with tunable mechanical properties and complex architectures, benefiting from the possibility of heat-assisted DLP in printing resins with high viscosity at room temperature.^142^

Moreover, PEGDA supports bioprinting technology by manufacturing biodegradable scaffolds for cell culture support, which holds significant potential in tissue engineering. For example, 2PP has been used to prepare hydroxyapatite-PEGDA scaffolds with various geometries and pore sizes.^143^ These scaffolds, functionalized with epidermal growth factor covalently connected to hydroxyapatite-gelatin methacrylate, exhibit a growth potential increase of up to 177%. Overall, the application of PEGDA in photopolymerization 3D printing offers a flexible and customizable material platform for developing biomedical scaffolds and customized drug delivery systems.

### 3.2 Biodegradable Photopolymers

#### 3.2.1 Poly(ε-caprolactone)-Based Photopolymers

Poly(ε-caprolactone) (PCL) is an FDA-approved biodegradable polymer known for its hydrophobic aliphatic polyester structure synthesized via ring-opening polymerization (ROP), which is highly compatible with biological systems.^144,145^ PCL’s compatibility with various drugs enables uniform distribution within the formulation matrix, and its long-term degradation supports drug release over several months.^146^

The hydrolysis rate, mechanical properties, and rheological properties of PCL can be tailored by copolymerization with other monomers or by incorporating ester bonds, such as lactide or glycolide, which hydrolyze more readily than typical ester bonds in PCL.^147^ Modulating PCL’s molecular weight, morphology, and structure allows customization for different drug delivery requirements, such as adjusting drug release rates and enhancing drug stability.^148^ PCL’s excellent biocompatibility, low immunogenicity, and superior molding capabilities make it widely applicable in fields like tissue engineering scaffolds^149,150^ and drug release carrier.^151,152^

In the field of photopolymerization 3D printing, PCL and its derivatives are also widely applied.^153,154^ Introducing photopolymerizable groups like acrylate and vinyl into PCL chains creates photosensitive materials that form a cross-linked network upon exposure to UV or visible light.^153^ The resin’s viscosity and mechanical properties can be adjusted by using different monomer composition or resin formulation.^155^ For instance, PCL diols were synthesized via ROP of ε-caprolactone with diethylene glycol as an initiator and Sn(Oct)_2_ as a catalyst.^155^ Polyurethane acrylates were developed using isophorone diisocyanate, PCL diol, and 2-hydroxyethyl acrylate to create a photocurable resin for DLP 3D printing technology, offering flexibility, biocompatibility, and degradability. To reduce resin viscosity and adjust mechanical properties, different ratios of PEGDA and PPG were added.

To decrease the resin viscosity and adjust mechanical properties, varying ratios of PEGDA and polypropylene glycol (PPG) were added to prepare different resin formulations.^155^ Utilizing the DLP 3D printing technique allows for the fabrication of complex structures with reduced repetition. These resins hold significant promise for tailored tissue engineering and various biomedical applications. Adding chitosan to PCL-based photopolymerization resin mitigates PCL’s hydrophobicity, enhancing cell adhesion and differentiation.^156^ Three-armed hydroxyl-terminated PCL oligomers were synthesized by the ring-opening polymerization of ε-caprolactone monomers, followed by methacrylation using methacrylic anhydride. Paunovic and colleagues copolymerized D,l-lactide (DLLA) and CL,^157,158^ resulting in amorphous copolymers with lower viscosity than homopolymers of the same molecular weight. Considering that branched polymers typically exhibit lower viscosity, higher cross-linking density, and better mechanical properties than linear polymers, a series of four-arm poly(DLLA-*co*-CL) polymers were synthesized and subsequently functionalized with methacrylates for photopolymerization to prepare airway stents.^157^ PCL’s longer degradation compared to polylactic acid (PLA) or polyglycolic acid (PGA) makes it suitable for long-term drug delivery devices with a half-life extending beyond one year.^159,160^

#### 3.2.2 Polylactic Acid-Based Photopolymers

PLA is another representative biodegradable polymer widely used in the field of 3D printing.^161^ The primary method for processing PLA in 3D printing is FDM, however, the printed products often suffer from rough surfaces, low precision, slow printing speeds, and low efficiency.^162^ Modifying PLA with photo-cross-linkable groups enables its 3D printing via vat photopolymerization. Melchels et al. prepared porous poly(D,l-lactide) constructs using SLA for the first time, using ethyl lactate as a nonreactive diluent.^163^ These photo-cross-linked networks exhibited good mechanical properties and cell adhesion, with proliferation rates comparable to high molecular weight poly(D,l-lactide) and tissue culture polystyrene. Jansen et al. prepared photo-cross-linked networks by copolymerizing fumaric acid monoethyl ester (FAME) end-functionalized, three-armed poly(d,l-lactide) (PDLLA) oligomers with N-vinyl-2-pyrrolidone (NVP) as a diluent and comonomer, resulting in networks with high gel contents and tunable hydrophilicity.^124^ Using stereolithography, these networks were used to create predesigned biodegradable tissue engineering scaffolds with optimized pore architecture and tunable material properties. Jašek et al. investigated the curing characteristics and thermomechanical properties of curable alkyl lactates based on PLA and poly(3-hydroxybutyrate) (PHB).^164^ Methacrylated alkyl lactates were found to exhibit suitable apparent viscosity for SLA 3D printing applications.

Due to the high glass transition temperature (*T*_g_) and high viscosity of PLA resins, it is rather challenging to print PLA photopolymers with high molecular weights.^161^ In general, to develop PLA-based copolymers remains the efficient way for vat photopolymerization. Felfel and colleagues utilized 2PP technology to fabricate scaffolds from poly(D,l-lactide-*co*-ε-caprolactone) copolymer with varying ratios of LA and CL.^165^ They produced 3D scaffolds with controlled porous architecture, defined microstructure, and adjustable degradation properties. Similarly, Paunovic et al. reported on the 4D printing of biodegradable shape-memory elastomers using poly(D,l-lactide-*co*-trimethylene carbonate) methacrylates with various monomer feed ratios, enabling adjustable transition points at physiological temperatures.^33^ These materials retain their deformed shape at room temperature and exhibit efficient shape recovery at 37 °C, along with cytocompatibility and biodegradability under physiological conditions. Furthermore, they achieved 4D-printed shape-memory drug-eluting devices with tunable drug-release kinetics using DLP printing. The photopolymer formulation can affect the drug release kinetics when hydrophobic or hydrophilic drug molecules are directly incorporated in the 3D printing resin.

#### 3.2.3 Poly(trimethylene carbonate)-Based Photopolymers

Poly(trimethylene carbonate) (PTMC) is an amorphous, flexible, and biodegradable polymer that can be functionalized with photo-cross-linkable units (e.g., MA groups) for photopolymerization.^166,167^ 3D printed PTMC networks exhibits good mechanical properties and biocompatibility, making them suitable for manufacturing various biodegradable scaffolds,^168^ implants,^169^ and medical devices.^170^ PTMC can also be combined with other synthetic polymers, such as PDLLA, PCL and PEG, to achieve tunable mechanical properties and a wider range of applications.^171^ High molecular weight PTMC degrades via surface erosion, while low molecular weight PTMC degrades more rapidly and uniformly. Photopolymerization of MA-functionalized PTMC oligomers yields tough and tear-resistant networks, with strength and toughness increasing with the molecular weight of the polymer used.^172^

For photopolymerization-based 3D printing, PTMC can form hybrid networks via the synthesis of copolymers, enabling control over resin viscosity and cross-linking network properties, including mechanical performance, degradation behavior, and cellular responses in vitro and in vivo.^169,173^ Kwon and colleagues photopolymerized various microstructures based on TMC liquid prepolymers using a custom-designed micro-SLA system to create multineedle microstructures as prototype models for sustained drug release in diseased tissues.^174^ Wang and co-workers designed 12 different PTMC-based multiblock copolymers with varied compositions or chain lengths by introducing poly(propylene fumarate) (PPF) blocks, and used them for projection micro-SLA (PμSL) printing.^175^ Their research demonstrated that the flexibility of copolymer chains positively correlates with the PTMC fraction. Schüller-Ravoo and colleagues utilized a photo-cross-linkable resin based on PTMC macromers to fabricate designed flexible and elastic network structures using stereolithography.^176^ These hydrophobic networks exhibited excellent physical properties and compatibility with human umbilical vein endothelial cells, designed as three-dimensional microvascular scaffolds with open channels to ensure efficient cell nourishment in large tissue volumes.

#### 3.2.4 Poly(propylene fumarate)-Based Photopolymers

Poly(propylene fumarate) (PPF) is another widely used biodegradable polymer, known for its vinyl groups on the backbone that makes it naturally photo-cross-linkable.^177^ First reported in 1994, PPF has been extensively investigated as a scaffold material for vascular stents,^178^ blood vessels,^179^ nerve grafts,^180^ and bone tissue engineering.^181,182^ Over time, improvements in synthesis methods have enhanced PPF’s applications.^183^ Becker and co-workers developed a novel synthesis method via ring-opening polymerization (ROP), which afforded PPF with controlled molecular weight and high-fidelity end groups. The polymers can be further isomerized into 3D printable PPF, facilitating the production of thin films and scaffolds suitable for postpolymerization and postprinting modification with bioactive agents.^184^

Recent studies have shown that by micro-SLA, precise scaffolds with controlled microstructures can be fabricated by adjusting the PPF viscosity through heating and incorporating diethyl fumarate (DEF).^185,186^ These scaffolds are suitable for tissue engineering applications. For instance, bone morphogenetic protein-2 (BMP-2)-loaded scaffolds combining PPF/DEF photosensitive polymers with a micro-SLA system and BMP-2-loaded microspheres can gradually release growth factors.^187^ By adjusting manufacturing parameters such as PPF molecular weight^188^ and PI content,^182^ degradation and mechanical properties can be effectively controlled. Higher molecular weights and photoinitiator concentrations result in slower degradation due to increased polymer chain length and cross-link density. Additionally, increasing the energy of photocuring significantly enhances the elastic modulus.^189^

Adjusting the concentration of photoinitiators allows control over the hardness and cross-linking density of the polymer, influencing its degradation behavior.^185^ This capability is particularly advantageous for applications requiring prolonged drug release, as it helps regulate the rate and duration of drug release to meet clinical needs. Choi et al. utilized PPF scaffolds as drug delivery matrices for the anticancer drug doxorubicin loaded with iron oxide nanoparticles or manganese oxide nanoparticles and measured the drug release under physiological conditions using MRI and optical imaging.^191^ They observed the slow release of drug molecules over several hours to days.

#### 3.2.5 Poly(glycerol sebacate)-Based Photopolymers

Poly(glycerol sebacate) (PGS) is a biodegradable polymer known for its good biocompatibility and processability.^192^ However, its chemical cross-linking requires harsh conditions (>80 °C, < 5 Pa) and extended reaction times (typically >24 h), limiting its application in direct polymerization within tissue or with temperature-sensitive molecules.^193^ Alternatively, PGS can be functionalized with photo-cross-linkable groups (e.g., acrylates) to enable the photopolymerization. Nijst et al. developed poly(glycerol sebacate acrylate) (PGSA), which can undergo rapid photo-cross-linking at room temperature, forming networks with tunable mechanical properties and degradation profiles.^193^ Incorporating PEG-DA with PGSA further allows control over mechanical properties and swelling behavior in aqueous environments. In vitro studies demonstrate the biocompatibility of these networks. Wang et al. successfully printed PGSA into complex network structures using DLP,^194^ mimicking the dual-network structure found in nature with interconnected segments of varying mechanical properties. Chen et al. copolymerized PCLDA and/or PEGDA with PGSA to form biodegradable copolymers, resulting in network polymers with tunable mechanical properties and significantly higher degradation rates.^195^ Using SLA technology, these photopolymerizable, biodegradable copolymers can be used to fabricate scaffolds with varying mechanical properties, enhancing their potential for applications in soft tissue engineering. Singh et al. evaluate poly(glycerol sebacate methacrylate), a photopolymerizable formulation derived from PGS, for peripheral nerve repair, demonstrating its efficacy in fabricating nerve guidance conduits via micro-SLA.^196^

#### 3.2.6 Poly(β-amino ester)s-Based Photopolymers

Poly(β-amino ester)s (PBAEs) are biodegradable cationic polymers first synthesized by Chiellini in 1983.^197^ They are easily produced via a one-step reaction coupling β-amino or bis(secondary amine) with diacrylate, requiring no further purification to remove byproducts.^198^ PBAEs have applications in protein delivery and gene therapy, forming hydrogels and nanoparticles.^199−203^ PBAEs exhibit tunable degradation behavior due to their diverse chemical compositions.^200,204^ For example, Louzao et al. screened a library of PBAE copolymers to find a 3D-printable formulation for subcutaneous implantation of paroxetine hydrochloride, enabling release from 253 formulations.^205^

PBAEs have also been used to fabricate biodegradable scaffolds tailored to specific requirements, providing structural support and facilitating new tissue formation during gradual degradation. We recently synthesized a series of PBAE photopolymers suitable for DLP 3D printing, and one formulation using dopamine and PEGDA was selected to prepare wound dressings, demonstrating promotion of wound healing and personalized wound care.^206^ By adjusting the formulation of PBAEs, their controllability and biocompatibility can be optimized, making them ideal for manufacturing complex medical devices. Additionally, PBAEs enable the fabrication of microscale drug delivery systems with precise shapes and structures using photopolymerization 3D printing, offering opportunities for targeted and controlled oral delivery.^122^ In addition, there are also other synthetic biodegradable photopolymers developed for vat photopolymerization, such as poly(1,12-dodecamethylene citrate) (PDC),^207^ poly(glycerol-dodecanoate) (PGD),^208^ and aliphatic polycarbonate,^209,210^ with potential for customized drug delivery systems.

#### 3.2.7 Naturally Derived Biomaterials

In addition to synthetic polymers, there are various biopolymers or naturally derived biomolecules available for vat photopolymerization, when functionalized with suitable photo-cross-linking groups. Vegetable oil (VO) is a common source of biobased resins, modified through chemically processes involving double bonds, epoxides, or (meth)acrylic esters to form thermosetting networks via photo-cross-linking.^211^ Guit et al. introduced epoxy soybean oil methacrylate, containing 74–83% biobased components and commercial biobased diluents, applied in DLP.^212^ Polyurethane-modified epoxy soybean oil can also be blended with acrylic esters for SLA, forming interpenetrating networks.^213^ Research into VOs-based 3D printing products, particularly in biomedicine, has focused on shape memory scaffolds and implants. Danish et al. used micro-SLA with acrylated epoxidized soybean oil,^214^ demonstrating shape memory effects with rapid recovery and high fixation rate, suitable for tissue scaffolds.

Lignin, the second most abundant natural polymer and the only one composed predominantly of aromatic hydrocarbons, is a highly renewable feedstock.^215^ Wang et al. demonstrate that lignin-derived dendritic colloidal materials (DCMs) significantly enhance the fidelity and mechanical properties of PEG-based hydrogels in DLP 3D printing by creating a dual-continuous morphology.^216^ The combined effects of light absorption and free radical reactivity of lignin-DCMs allow for high geometric fidelity and structural complexity in the photopatterning of dilute PEG hydrogels (5–10%), increasing their toughness 6-fold compared to pure PEG hydrogels.

Vanillin is a common organic compound widely used in the food and flavoring industry, and in recent years, its application in SLA has been noted for its thermal stability and recyclability.^217^ Combining vanillin methacrylate with glycerol dimethacrylate yields 3D printed products with *T*_g_ of 153 °C and Young’s modulus of 4900 MPa.^217^ Cage-like thiolated vanillin can be used to modify gelatin hydrogels, which are untangled using two-photon light to create reactive thiol groups. Sequentially immobilizing barnase and streptavidin via two-photon chemistry and complexing them with fusion proteins, produced biologically active 3D patterned hydrogels.^218^

Natural polymers like gelatin, collagen, agarose, and chitosan are inherently biodegradable and biocompatible, making them suitable as customized drug delivery carriers. Gelatin methacrylate (GelMA) is notable for creating biocompatible 3D structures, drug screening, disease modeling, and tissue repair.^219−221^ Introducing hydroxyapatite (HAp) to GelMA networks improves mechanical properties and osteogenic differentiation, rendering the GelMA/HAp porous composite scaffold, mimicking bone matrix and customizable for DLP printing, with tremendous potential for bone tissue repair.^222^ Song et al. fabricated a hierarchical biomimetic microporous hydrogel composite scaffold by synthesizing GelMA and methacrylic anhydride silk fibroin (SilMA), creating GelMA/SilMA inks, and incorporating HAp using an aqueous two-phase emulsification method.^223^ In vitro and in vivo experiments demonstrated that the resulting M-GSH scaffolds significantly promoted cell adhesion, proliferation, and osteogenic differentiation. Zhong et al. used a DLP-based bioprinting system to prepare microscale hydrogel scaffolds composed of GelMA and hyaluronic acid glycidyl methacrylate. These scaffolds supported the encapsulation and viability of primary rabbit limbal stem/progenitor cells.^224^ Lin et al. utilized a methacrylated gelatin (mGL) solution and visible light-based SLA to create three-dimensional hydrogel scaffolds containing the BMP-2 gene and green fluorescent protein reporter gene for bone formation research.^225^ The results demonstrated that these gene- and cell-activated gelatin scaffolds significantly promoted BMP-2 release and bone formation both in vitro and in vivo, indicating their potential for treating bone defects.

Collagen, found in connective tissues, is essential for tissue regeneration.^226^ It is used in biofabrication, tissue engineering, and regenerative medicine.^227^ Du et al. created a fluorescent bioink for DLP 3D bioprinting, blending collagen with cyanine IR-780 and high molecular weight type I collagen, improving cell compatibility and printing resolution.^228^ Wu et al. developed a ColMA-based bioink suitable for DLP 3D bioprinting, with lithium phenyl (2,4,6-trimethylbenzoyl) phosphinate and purpurin (PA) as the PI and cross-linking agent. The rapid photopolymerization of the bioink, combined with the introduction of PA, enhanced performance and cell compatibility, with ColMA-PA demonstrating excellent potential as a biomaterial in tissue engineering.^229^

Chitosan, derived from chitin and exoskeletons of crustaceans, is known for its biocompatibility and antibacterial properties.^230^ By photopolymerization 3D printing, chitosan-based hydrogels have been manufactured to create complex, porous structures promoting cell adhesion for tissue repair. He et al. used a hybrid bioink of methacryloyl-modified chitosan and acrylamide for DLP 3D printing, producing hydrogel with enhanced compression strength and elasticity.^231^ However, the crystalline structure of chitosan, sustained by intermolecular and intramolecular hydrogen bonds, restricts its solubility, creating challenges when it is combined with other photopolymerizable synthetic polymers. To tackle this issue, N-succinyl modification was employed to improve solubility. This strategy enables photopolymerization via 2PP, and maintains biocompatibility, making it suitable for creating precise microstructures for cell cultivation.^232^ Bozuyuk et al. utilized chitosan with 70% methacrylation to prepare microswimmers using two-photon printing for the delivery of the chemotherapeutic drug doxorubicin.^39^

Alginate is a widely used biomaterial with excellent biocompatibility, low toxicity, low cost, and a convenient gelation process, making it ideal for bioprinting.^233^ Alginate can be modified with photo-cross-linkable groups such as dienes and acrylate groups to enable photopolymerization.^234^ For example, sodium alginate and 2-aminoethyl methacrylate can be reacted in the presence of 1-ethyl-3-(3-(dimethylamino)propyl) carbodiimide hydrochloride and *N*-hydroxysuccinimide to prepare photo-cross-linkable alginate macromolecules.^235^ The methacrylated alginate was then photo-cross-linked under UV light with Irgacure D-2959 as PI. Valentin and colleagues developed a 3D stereolithographic printing method for a ionically cross-linked hydrogel.^236^ In the presence of insoluble divalent cation salts, these alginate hydrogels can be printed using SLA technology, and adjustments in degradation kinetics, pattern fidelity, and mechanical properties can be achieved by altering the hydrogel formulation. This study highlights the multifunctionality of alginate hydrogels, which can be utilized in various applications, such as microfluidic channel fabrication and guiding cell migration.

## 4 Drug Loading Methods and Release Manners

**Figure 4:**
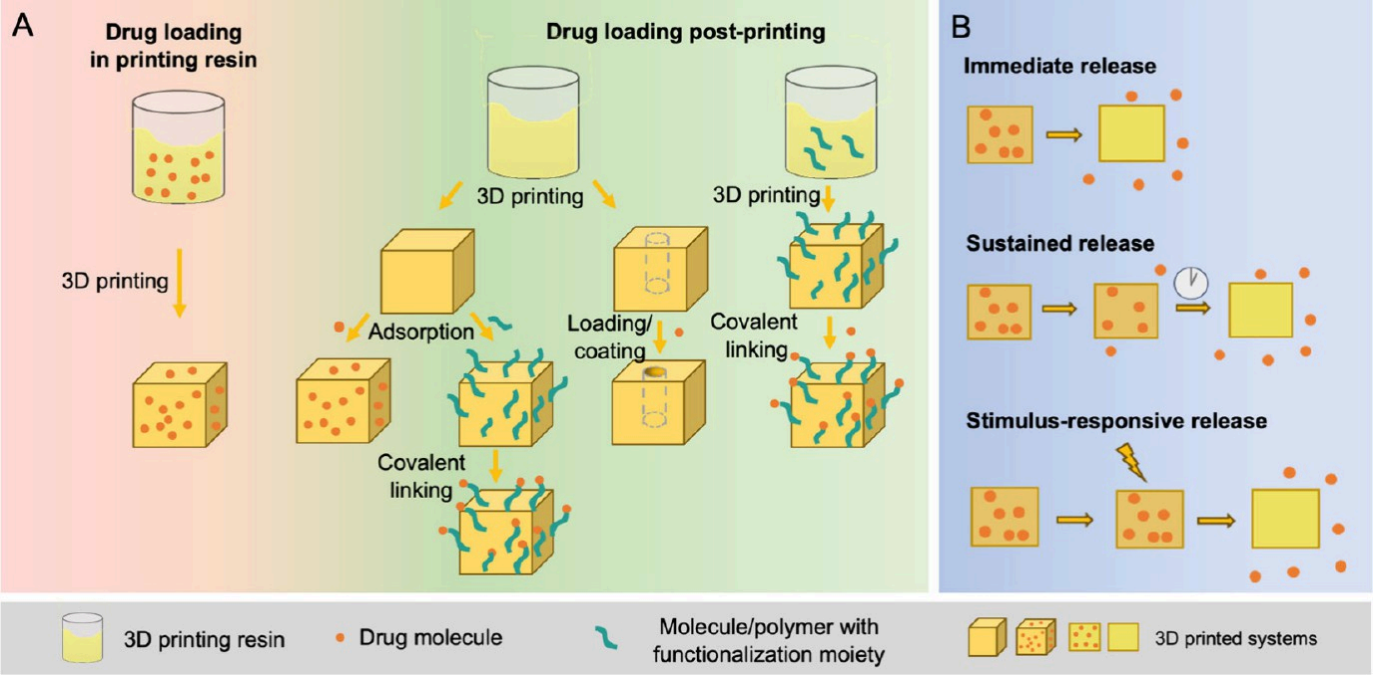
Schematic diagram of drug loading methods (A) and drug release manners (B) in 3D printed drug delivery systems.

Based on aforementioned biocompatible and biodegradable materials, photopolymerization 3D printing materials have been employed to manufacture various drug formulation and delivery systems such as tablets,^56^ microneedles,^237^ microrobots,^238^ and functional implants and devices.^239^ The appropriate methods for drug loading are crucial for the controlled drug release from the 3D printed systems. Direct drug loading in the printing resin is the simplest method of incorporating drugs, allowing for uniform distribution within the printed object. It is particularly suitable for the drug molecules that are not degraded by UV light exposure (Figure 4A, left). Postprinting drug loading allows for maintaining the integrity of drugs by incorporating them after the photopolymerization process, while covalent drug conjugation securely attaches the drug to the polymer matrix, ensuring a controlled release (Figure 4, right). Nonetheless, postprinting loading may pose challenges in achieving uniform drug distribution, whereas covalent conjugation, while offering stability, might involve complex chemical processes that could potentially affect drug activity. Selecting the optimal method depends on the specific requirements of the drug delivery system and the nature of the drugs involved.

### 4.1 Drug Loading Methods

#### 4.1.1 Physical Drug Loading

In a physical way, drugs can be incorporated into photopolymerizable 3D printing materials either by direct dissolution or dispersion in the liquid resin, or by loading drugs onto the device postmanufacture through absorption, adsorption, or filling.^240^

##### 4.1.1.1 Drug Loading in Printing Resin

The straightforward physical approach involves directly mixing the drugs into the liquid resin, where it can be completely dissolved or evenly dispersed by magnetic stirring at room temperature in a resin composed of PI and photopolymers.^241,242^ After printing, the drugs are physically encapsulated within the cross-linked polymer network and is released through diffusion as the matrix swells or degrades in the dissolution medium. For example, Wang et al. used SLA printing to manufacture drug-loaded tablets by dissolving paracetamol in a photosensitive resin solution composed of PEGDA and PEG.^56^ While multidrug therapy presents a significant prescription challenge, administering each drug separately can be inconvenient and may lead to medication and compliance issues. Martinez et al. address this by using SLA to fabricate composite drug pellets containing six active ingredients, including paracetamol, caffeine, naproxen, chloramphenicol, prednisolone, and aspirin.^136^ They dissolved the drugs and PI in a liquid resin, stirred until completely dissolved, and then poured the solutions into resin trays for printing.

Key considerations when employing this method include solubility, compatibility, stability, and the desired release profile.^240^ Addressing these factors is essential to achieve uniform drug distribution, preserve the integrity and functionality of the printed product, and ensure its therapeutic efficacy and safety.^243^ The drugs can also be dispersed in the resin via a secondary phase, such as insoluble drug particulates, drug-loaded nano- or microparticles, or as an emulsion.^240^ For instance, the BMP-2 growth factor can be loaded into microspheres and then mixed with photosensitive polymers for microstereolithography printing to prepare 3D scaffolds. In vivo testing has shown that BMP-2 releasing scaffolds promote bone formation.^187^

##### 4.1.1.2 Drug Loading Postprinting

Despite the convenience, fabricating drug delivery systems via photopolymerization 3D printing necessitates careful consideration to prevent adverse reactions between photopolymers and drugs.^242^ These interactions can degrade or alter the active drug molecules, diminishing therapeutic efficacy. Some drugs have limited solubility or degrade when exposed to high temperatures.^244^ For devices printed with drug-free resins, drugs can be incorporated using adsorption-based techniques, such as immersion and spraying.^9,31^ Alternatively, drugs can be absorbed into the polymer network by swelling the device in a concentrated drug solution or by loading drugs into printed hollow structures. For example, Uddin and colleagues used SLA printing to fabricate microneedles, followed by inkjet coating of a cisplatin polymer layer onto the microneedle surface. Embedding the drug within a rapid-dissolving layer composed of hydrophilic PEG–PVP-PEG polymers facilitated the rapid delivery of the hydrophobic drug cisplatin into the epidermis.^32^ Vaut et al. employed DLP to fabricate reservoir devices for oral drug delivery.^245^ Their design incorporates drugs into reservoirs with anchor-like surface structures to enhance mucosal adhesion and intestinal retention. Compared to untextured controls, these textured surfaces doubled the mucosal adhesion of the device. In our previous research, we designed oral patches mimicking octopus suction cups, achieving drug delivery by directly incorporating drugs into the printed structures.^37^ While postloading adds additional manufacturing steps, it can prevent potential drug degradation during preprinting or printing processes.

#### 4.1.2 Covalent Drug Conjugation

##### 4.1.2.1 Drug Loading in Printing Resin

Instead of physical mixing with 3D printing resin, the drug molecules can be first functionalized and then cross-linked to 3D printed object via covalent bonds. Similar to the prodrug approach, covalent bonding enables sustained drug release by reducing the likelihood of burst release and allows for precise control over the release rate.^246^ By designing different linkers or conjugation methods, the release profile of the drug can be effectively regulated.^247^ He et al. functionalized ibuprofen with 2-hydroxyethyl acrylate via a cleavable ester bond, formulating it for inkjet 3D printing.^247^ This method utilizes a reactive prodrug that polymerizes during printing to form drug-attached macromolecules, and by adjusting hydrophilicity, achieving a drug loading of up to 58 wt %. Para-nitrobenzyl is a widely used photocleavable functional group. Studies have shown that para-nitrobenzyl derivatives containing *N*-hydroxysuccinimide ester (NHS) and alkyne groups are highly effective in controlled release. The NHS group selectively reacts with amino groups (NHS-amine coupling), while the alkyne group reacts with azide groups (copper(I)-catalyzed click reaction). Bozuyuk et al. present a photocleavage-based light-triggered drug delivery system that utilizes o-nitrobenzyl linker molecules for the controlled release of DOX through selective chemical reactions.^39^ Using 2PP, they successfully prepared DOX-functionalized microswimmers. When irradiated with light at a wavelength of 365 nm, the o-nitrobenzyl linkers undergo selective bond cleavage, enabling the release of DOX from the microswimmers. This successful conjugation of DOX significantly improves drug delivery efficiency, as evidenced by increased fluorescence emission in comparison to the control group. Zhang et al. developed a novel photopolymerized 3D printing scaffold using Pt(IV) prodrug initiators for postsurgical tumor treatment.^248^ The Pt-GelMA scaffold was fabricated from microfluidic 3D printing of GelMA bioinks, employing a Pt(IV)-induced photo-cross-linking process without additional PI or chemotherapy drugs. Activated by light, the Pt(IV) prodrug initiates scaffold polymerization, generating cytotoxic Pt(II) species for tumor therapy and tissue repair. This method avoids the cumbersome use of traditional chemical initiators and cross-linkers in 3D scaffold preparation, demonstrating promising clinical applications.

##### 4.1.2.2 Drug Loading Postprinting

Covalent drug conjugation were also utilized for 3D printed drug delivery devices. In these cases, drug loading postprinting involves chemically attaching drug molecules to the surface or structure of a printed device. Postprinting drug loading can be achieved through various chemical reactions, such as click chemistry, amide bond formation, or esterification, which form strong covalent bonds between the drug and the polymer matrix of the printed device.^249^ Wilson and colleagues utilized PPF to print films and scaffolds with functional moieties for postpolymerization and postprinting modification.^184^ The authors employed copper-mediated azide–alkyne cycloaddition to attach small molecule dyes and the cell-adhesive peptide GRGDS onto the surface, demonstrating the potential for functionalizing 3D-printed materials with bioactive molecules. Wan et al. utilized DLP 3D printing technology for photopolymerization to manufacture peptide-containing objects and studied their release kinetics.^250^ They first synthesized a disulfide-functionalized bis(acrylamide) and incorporated it into a printing ink based on hydroxyethyl acrylate to enhance its solubility. After printing, thiol groups were introduced using tris(2-carboxyethyl) phosphine, enabling thiol–disulfide exchange with disulfide-containing peptides like lanreotide. This method effectively enables the fabrication of complex geometries with peptide covalent binding capabilities.

### 4.2 Drug Release Manners

By utilizing different drug-loading methods and materials, 3D printed drug delivery systems can achieve various release profiles, such as immediate release, sustained release or stimuli-responsive release (Figure 4B). The selection and design of these release profiles depend on specific therapeutic needs and drug properties.

#### 4.2.1 Immediate Release

Immediate drug release is particularly suitable for situations requiring quick relief, such as acute pain or elevated blood sugar levels.^251^ By releasing quickly and achieving maximum plasma concentration, immediate release drugs can reduce fluctuations of drug levels within the body, thereby more effectively controlling symptoms.^252^ General strategies to achieve fast drug release include using highly soluble drug forms or salts that rapidly dissociate in body fluids.^253^ Common techniques involve immediate-release tablet formulations, where excipients facilitate quick disintegration and dissolution.^254^ Injectable forms, particularly intravenous injections, offer the fastest onset by delivering the drug directly into the bloodstream.^255^ Additionally, advanced technologies like nanoemulsions and fast-dissolving films can further enhance the speed of drug release, ensuring swift relief for acute conditions.^256,257^

Economidou et al. used SLA printing to fabricate microneedle arrays from commercial resin, which were then coated with insulin and the sugars sorbitol, mannitol, and trehalose.^258^ In vivo experiments on diabetic mouse models demonstrated that insulin-coated microneedles effectively controlled blood glucose levels within 60 min. In vitro studies showed that 90–95% of the insulin was released within 30 min. Compared to subcutaneous injections, the 3D printed microneedle arrays provided a rapid reduction in blood glucose levels and maintain these levels for a longer duration. D‘hers et al. designed an SLA-printed emergency insulin injection device called rapid reconstitution package (RRP).^259^ This device was a prefilled cartridge capable of long-term storage, on-demand reconstitution, and delivery of therapeutic drugs using standard syringes. Ropinirole hydrochloride, a dopamine agonist used to treat Parkinson’s disease and restless legs syndrome, was formulated into a UV inkjet 3D printed tablet by Clark et al. This immediate-release formulation released 89% of the drugs within 4 h.^260^ Xu et al. investigated the effects of shape on the release of ibuprofen-loaded particles manufactured using SLA printing.^35^ The study found that altering the size of the printed particles, rather than adjusting the formulation, could modulate the drug release rate. Small particles (1–2 mm) exhibited release behavior independent of PEG 400, indicating that drug release could be controlled through size adjustment. Additionally, printing formulations without hydrophilic binders enhanced the adaptability mechanical performance of the produced doses.

#### 4.2.2 Sustained Release

Sustained release mode is designed to gradually release the active ingredients into the bloodstream over an extended period, thereby prolonging the drug’s action and effectiveness.^261^ This type of drug delivery is often employed to reduce dosing frequency, enhancing patient convenience and compliance.^262^ By releasing drug molecules slowly, sustained release formulations help maintain stable blood drug concentrations, minimizing abrupt fluctuations and reducing the likelihood of adverse effects.^261^ Certain medications, such as anti-inflammatories, antibiotics, or analgesics, exhibit improved efficacy when released gradually, enhancing therapeutic outcomes.^263^ Additionally, sustained release drugs can attenuate the peak concentrations, thereby reducing potential adverse impact on organs like the cardiovascular and digestive systems.^264^ General strategies to realize drug sustained release involve various techniques to control the rate at which a drug is released into the body, ensuring a consistent therapeutic effect over an extended period.^264^ These techniques include the use of polymer-based matrices that slowly degrade or swell, releasing the drug gradually. Encapsulation of drugs in microspheres or nanospheres allows for a controlled diffusion process.^265^ Additionally, embedding drugs in hydrogels or liposomes can modulate release rates through osmosis or membrane permeability.^266^

Chen et al. utilized AA as a monomer and poly(ethylene glycol) dimethacrylate (PEGDMA) as a bifunctional cross-linker, incorporating acrylicized hyperbranched polyester multifunctional cross-linkers to inhibit premature drug release. Tablets printed using DLP in simulated gastrointestinal fluids exhibited sustained release of the model drug 5-fluorouracil for over 24 h.^267^ Xu et al. developed a bladder device based on elastomers, fabricated using SLA printing, for intravesical drug delivery. This device integrates localized bladder therapy with prolonged drug exposure,^50^ enabling sustained drug release for up to 14 days.

Modifying the shape of drug delivery devices is another strategy to control drug release kinetics. Janusziewicz et al. validated that formulations printed using digital light synthesis influence the swelling, uptake, and in vitro release of two model drugs (β-estradiol - hydrophobic, and 2-fluoro-2′-deoxyadenosine - hydrophilic). The authors found that sustained drug delivery is driven by the geometry of the parts.^89^ For instance, the hydrophilic antiretroviral drug 4′-ethynyl-2-fluoro-2′-deoxyadenosine showed sustained release for over 70 days in simulated vaginal fluid. In another study, SLA printing was used to manufacture oleuropein (OLE) sustained-release gel blocks, achieving customized release behavior based on the surface area or volume of the printed devices.^269^ After 6 h, approximately 80% of OLE was released from square gel blocks, while circular and hexagonal structures released about 70% of OLE. Circular structures exhibited approximately 45% release after 6 h, reaching 90% after 24 h.

Martinez and colleagues employed SLA printing to fabricate tablets composed of dispersed acetaminophen within PEG.^137^ Various geometric shapes, including cubes, disks, pyramids, spheres, and rings, were generated with constant surface area (SA) or constant surface area-to-volume ratio (SA/V). Dissolution tests indicated that tablets with a constant SA/V ratio released the drug at the same rate, while those with a constant SA released the drug at different rates. For tablets with an SA/V ratio of 0.5, ring-shaped tablets released only about 20% of the acetaminophen after 10 h. The development of sustained release drugs and the exploration of 3D printing techniques for customized drug delivery systems offer promising avenues for enhancing therapeutic efficacy and patient convenience in medication administration.

#### 4.2.3 Stimulus-Responsive Release

Stimulus-responsive release has been achieved with advanced drug delivery systems that realize drug release in response to external stimuli such as light, temperature, pH.^270^ These systems offer several advantages over traditional controlled-release systems.^271^ They allow for precise control over drug release, adjustment of release rate, quantity, or timing based on specific stimuli, thus better meeting therapeutic needs.^272^ Additionally, stimulus-responsive systems are often reversible, allowing drug release to cease or slow down when the stimulus is removed, preventing drug overdose or excessive therapy.^273^ These systems also possess an intelligent capability to sense environmental changes and respond accordingly, facilitating smart drug release.^274^ General strategies for achieving responsive drug release rely on smart materials that respond to specific physiological or external stimuli, allowing precise control over drug delivery.^275^ These materials incorporate pH-sensitive linkers like hydrazone and acetal bonds, enzyme-sensitive linkers that are cleaved by specific enzymes, and redox-sensitive disulfide bonds.^276,277^ These mechanisms ensure targeted drug release in response to conditions such as pH variations in tumor microenvironments or enzymatic activity in diseased tissues.^278^

For example, light-responsive drug delivery systems can regulate the release of drug molecules based on photolysis. Typically, drug molecules are functionalized with photosensitive linkers, allowing for modifications at available chemical sites without compromising the therapeutic efficacy. Upon exposure to light, the photosensitive linker gets cleaved, releasing the drug molecule. Bozuyuk et al. utilized modified dextran-doxorubicin as a model for light-triggered drug release, coupling it to microswimmers via a neighboring nitrobenzyl linker.^39^ Under 365 nm light irradiation, the linker molecule cleaves selectively, releasing DOX. The microswimmers, fabricated using the TDLW, combine light-triggered drug delivery with magnetic propulsion, enabling precise and efficient on-demand medical tasks. Zhu et al. introduced a patch prepared via the DLP method, incorporating self-heating gold nanoparticles and ion-induced shape memory polymers (SMP).^279^ The gold nanoparticles generated heat through photothermal effects, enhancing drug penetration and controlling drug release. The ion-induced shape memory polymers change shape in response to ionic stimulation, forming microneedle structures that improve drug penetration efficiency when the patch contacts the skin. Sun et al. utilized DLP technology to fabricate a microneedle patch capable of responsive drug release.^280^ These microneedle patches incorporate two key design features: reversible shrink-swell behavior and NIR light-controlled drug release mechanism. By embedding graphene oxide (GO) into the microneedle base, researchers achieved NIR light-controlled drug release. GO exhibits excellent photothermal conversion efficiency, effectively generating heat under NIR irradiation at 808 nm. This photothermal effect in the microneedle base rapidly increases the temperature, thereby modulating the drug release rate from the patch. By adjusting the intensity and duration of NIR light exposure, researchers can precisely control the rate and amount of drug release.

In addition to light, pH or enzyme-triggered drug release systems are also widely designed. For example, Ceylan and colleagues developed a hydrogel microrobot swimmer that respond to matrix metalloproteinase-2 (MMP-2) for extracorporeal therapeutic and diagnostic tasks.^40^ High MMP-2 concentrations trigger rapid expansion of the hydrogel network, enhancing drug release. This microrobot, made using 2PP, contains iron oxide nanoparticles dispersed in methacrylated gelatin. Experiments with magnetic resonance imaging agents labeled with anti-ErbB 2 antibody-tagged magnetic nanoparticles demonstrated enzyme-triggered release, identifying ErbB2-marked SKBR3 cancer cells. Ceylan et al. also fabricated microswimmers using 2PP, composed of materials with magnetic iron oxide nanoparticles and human blood proteins.^281^ They used protease and pH changes to observe microswimmer swelling and evaluated stimulus-responsive release by incorporating the fluorescent small molecule CellTracker Deep Red dye. At a pH of 12, a rapid decrease in fluorescence indicated drug release. When treated with pancreatic protease, the microswimmers swelled rapidly, releasing the fluorescent drug into the environment. These examples illustrate the potential of stimulus-responsive release systems to enhance therapeutic efficacy, reduce side effects, and provide intelligent drug delivery solutions.

3D printing is also applied in the field of oral formulations to produce responsive release devices. Larush et al. developed a hydrogel formulation using DLP printing as a model for 3D printed controlled drug delivery systems.^282^ Hydrogel tablets with complex structures were printed using AA as the monomer and PEGDA as a cross-linker, resulting in a pH-responsive drug delivery system. For the model drug sulforhodamine B, the release percentages after 24 h were 65.7% from the hive structure and 12.3% from the box structure at pH 7.4, while at pH 1.2, they were 44.4% and 8.5%, respectively. Stimuli-responsive release systems hold promising prospects in various fields such as drug delivery, targeted therapy, and biosensors. Their application in triggered drug delivery devices allows for greater customization of therapeutic devices.

## 5 New Trends in Photopolymerization 3D Printing for Customized Drug Delivery

With the significant developments of 3D printing photopolymers and advanced photopolymerization techniques, numerous advanced drug delivery systems with customized functions have been developed. In oral delivery, photopolymerization 3D printing technology can be used to manufacture oral tablets or capsules with complex geometries and porous structures, enabling customized drug release.^283^ By adjusting printing parameters and material formulations, the release rate of the drug can be precisely controlled, enhancing therapeutic outcomes.^136^ Microneedles are a minimally invasive method for transdermal drug delivery, and 3D printing can produce precise, uniform, and structurally complex microneedle arrays.^284^ These microneedles can painlessly penetrate the skin barrier, delivering drugs directly to the target area, significantly improving drug absorption and efficacy. Additionally, photopolymerization 3D printing plays an important role in the manufacturing of implants and medical devices.^285^ Through personalized design and precise fabrication, implants that perfectly match the patient’s anatomical structure, such as bone repair scaffolds and drug-eluting stents, can be produced, providing more effective treatment options and better patient experiences. Microrobots represent another emerging application of photopolymerization 3D printing in drug delivery.^286^ 3D printing can be used to create small, precise, and multifunctional microrobots that can autonomously navigate within the body and deliver drugs to specific disease sites, achieving targeted therapy. Furthermore, photopolymerization 3D printing can also produce unconventional drug delivery devices, such as suction cup devices and detoxification scaffolds.^37^ These advanced applications are discussed in the following sections.

### 5.1 Oral Drug Formulations

Oral administration is a safe and convenient treatment method that plays a crucial role in enhancing patient compliance.^287^ With the advancement of technologies such as photopolymerization 3D printing, the field of oral drug delivery is witnessing new innovations. Oral administration is cost-effective and relatively inexpensive, making it widely adopted in clinical treatment.^288^

Oral tablets are the most common form of oral drug administration and a significant research focus in the field of photopolymerization 3D printing. This field has seen substantial advancements in the formulation of drugs and the customization of drug release profiles. For instance, Wang et al. used SLA to print prototype medications containing 4-aminosalicylic acid and paracetamol, creating drug-loaded tablets with specific sustained-release profiles.^56^ They achieved personalized dosing by adjusting the percentage of cross-linked polymers to modulate drug release kinetics.

**Figure 5:**
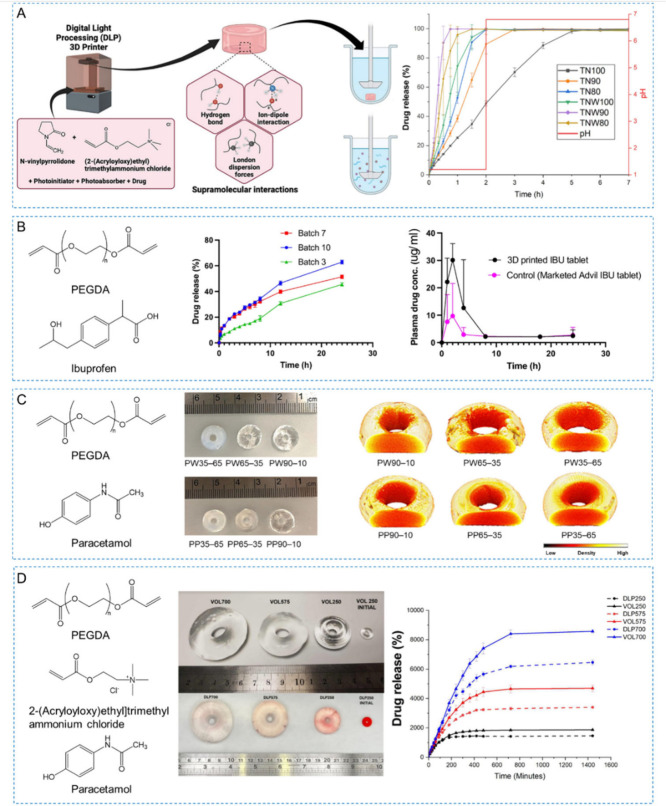
Photopolymerization 3D printing of oral drug formulations. (A) Fabrication of water-soluble drug carriers using DLP 3D printing. Reprinted or adapted with permission under a Creative Commons CC-BY from ref (289). Copyright 2023 International Journal of Pharmaceutics. (B) Enhanced drug release and pharmacokinetics of 3D-printed sustained-release ibuprofen tablets. Adapted with permission from ref (290). Copyright 2023 AAPS PharmSciTech. (C) Rotatory volumetric printing enabling the rapid production of personalized oral drug formulations. Reprinted or adapted with permission under a Creative Commons CC-BY from ref (109). Copyright 2023 International Journal of Pharmaceutics: X. (D) Fabrication of the drug-eluting devices by volumetric printing. Reprinted or adapted with permission under a Creative Commons CC-BY from ref (283). Copyright 2024 Research Square.

Recently, Ong et al. employed DLP 3D printing technology to fabricate water-soluble drug carriers using hydrophilic monomers [2-(acryloyloxy)ethyl] trimethylammonium chloride and NVP (Figure 5A).^289^ These carriers demonstrated efficient drug release rates, completely releasing the loaded drug (e.g., paracetamol) within 45 min to 5 h. By using dynamic supramolecular interactions between polymer chains instead of traditional cross-linking chemistry, they achieved the fabrication of linear polymer-based water-soluble structures. This approach enhances the versatility of drug release profiles and expands the scope of photopolymerization 3D printing in the pharmaceutical field. Mosley-Kellum et al. successfully utilized DLP 3D printing technology to fabricate sustained-release ibuprofen (IBU) tablets using materials such as PEGDA 700, water, IBU, and riboflavin (Figure 5B).^290^ By optimizing the formulation and printing parameters, these tablets demonstrated a drug release rate of over 70% within 24 h in vitro and significantly enhanced systemic absorption in vivo. Compared to commercially available ibuprofen tablets, the 3D-printed tablets showed higher peak plasma concentration and area under the curve values. In vitro-in vivo correlation studies indicated that the 3D-printed tablets could sustain the release of ibuprofen over 24 h. These innovations highlight the versatility of 3D printing in tailoring drug release behaviors to meet specific therapeutic needs.

3D printing technology overcomes the limitations of traditional methods for manufacturing tablets with multiple drugs, allowing for customized dosages and drug release profiles in complex formulations. Robles-Martinez et al. adapted a commercial SLA printer for their study, utilizing PEGDA as the photopolymerizable monomer and TPO as the PI.^136^ Each formulation was prepared by dissolving the drug and the PI in liquid PEGDA, with PEG300 included when applicable. This multiresin printing approach enabled the production of customized combination tablets containing six different active ingredients: acetaminophen, naproxen, caffeine, aspirin, prednisolone, and chloramphenicol. Through optimization of the printer platform, the process enables pausing, removal, and replacement of resin trays during printing, demonstrating adjustable physicochemical properties and varied drug release profiles.

On the technological front, volumetric printing has emerged as a promising method for fabricating oral dosage forms with responsive properties. As shown in Figure 5C, Rodríguez-Pombo et al. utilized paracetamol as the model drug, PEGDA with molecular weights of 575 and 700 as photoreactive monomers, PEG 300 as a nonreactive diluent, and LAP as the photoinitiator.^109^ Using tomographic volumetric printing, they successfully simultaneously manufactured torus and cylinder-shaped tablets loaded with paracetamol, with manufacturing times ranging from 12 to 32 s. By optimizing printing parameters such as rotation speed, light intensity, and exposure time, they achieved controlled drug release profiles by adjusting the ratios of photoreactive monomers and diluents. As shown in Figure 5D, Chan et al. conducted systematic comparison between volumetric printing and DLP technologies for fabricating oral dosage forms.^283^ The resin formulation included [2-(acryloyloxy)ethyl] trimethylammonium chloride (TMAEA) as the primary matrix monomer, PEGDA as the cross-linker, and paracetamol as the model drug, with LAP employed as the PI. Volumetric printing successfully created drug-eluting devices in a short time, exhibiting high water absorption and dynamic dimensional changes, with physicochemical properties similar to those of DLP-printed devices. Such advancements underscore the potential of volumetric printing in creating responsive tablets with tailored drug release kinetics. The summary of photopolymerization 3D printed oral drug formulations is presented in Table 1.

**Table 1:** Summary of Photopolymerization 3D Printed Oral Drug Formulations

Drug molecule	Drug delivery system	Materials	Drug loading mode	Drug release mode	3D Print Technology^ref^
**4-aminosalicylic acid and paracetamol**	Tablet	PEGDA	Direct dissolution	Modified release	SLA^56^
**Ibuprofen**	Tablet	PEGDA, PEG	Immersion	Sustained release	SLA^140^
**Ascorbic acid**	Tablet	PEGDMA	Direct dissolution	Modified release	SLA^291^
**Theophylline**	Tablet	PEGDA, PEGDMA	Direct dissolution	Modified release	DLP^82^
**5-fluorouracil**	Tablet	Acrylated hbpe, PEGDMA	Direct dissolution	Sustained release	DLP^267^
**Paracetamol**	Tablet	PEGDA	Direct dissolution	Modified release	volumetric 3D printing^283^
**Paracetamol**	Tablet	PEGDA	Direct dissolution	Modified release	DLP and tomographic volumetric 3D printing^109^
**Paracetamol**	Tablet	TMAEA	Direct dissolution	Modified release	DLP^289^
**Ibuprofen**	Tablet	PEGDA	Direct dissolution	Sustained release	DLP^290^
**Paracetamol, caffeine, naproxen, chloramphenicol, prednisolone and aspirin**	Multilayered polypill	PEGDA	Direct dissolution	Modified release	SLA^136^

### 5.2 Customized Microneedles

Topical and transdermal administration involve applying medications directly onto the skin or mucous membranes.^292,293^ They offer several advantages, including localized treatment, convenience, avoidance of first-pass metabolism, stable drug release, reduced side effects, noninvasiveness, and suitability for various medications.^294^ These characteristics make topical and transdermal administration important therapeutic options in clinical practice for treating various diseases and symptoms.^295^

Microneedle transdermal drug delivery systems have attracted widespread attention due to their noninvasiveness, improved drug delivery efficiency, stable release profiles, reduced drug dosage, and ease of use.^296,297^ By penetrating the skin’s stratum corneum, microneedles deliver drugs directly to superficial tissues or the bloodstream, enhancing drug absorption and bioavailability while minimizing side effects.^298^ To effectively deliver drugs, microneedles must meet several requirements. They must penetrate the stratum corneum, the outermost skin layer approximately 15 μm thick,^299,300^ which serves as a significant barrier to transdermal drug transport. Microneedles with high aspect ratios and small curvature radii require lower skin penetration force.^301,302^ Compared to individual needles, microneedle arrays can provide drug delivery or extraction over a wider area and at a higher rate.^302^ Consequently, high-resolution and multimaterial selection photopolymerization 3D printing has become the primary method for developing transdermal drug delivery microneedles, enabling the creation of precise, effective, and patient-friendly drug delivery systems, facilitating treatment for various diseases.

Skin cancer is a common skin disease, and localized treatment offers significant advantages.^303^ Using microneedles in the treatment of skin cancer allows direct delivery of anticancer drugs to the affected skin area, achieving local therapy without affecting surrounding healthy tissues.^304^ Through microneedle technology, drugs can be precisely released into the tissue surrounding tumor cells, thereby increasing local drug concentration, reducing systemic side effects, and enhancing the efficacy of anticancer treatment.^305^ Dacarbazine, an anticancer drug, is incorporated into a PPF mixture and then photopolymerized using a micro-SLA to prepare a microneedle array.^284^ This technique allows precise control over the size and length of the microneedles, ensuring minimal damage to the dermis and nerve endings during penetration. The drug encapsulated within the microneedles is released from the drug-loaded PPF matrix, and by adjusting the drug loading and molecular weight of the PPF monomer, the drug release rate can be regulated. Microneedles loaded with PPF present a potential therapeutic approach for treating skin cancer by directly targeting the lesion area, providing localized treatment without affecting surrounding healthy tissue. Similarly, cisplatin, another anticancer drug, was incorporated into the surface of an SLA 3D-printed microneedle array with a cross-shaped design.^32^ In vivo evaluation showed a rapid release rate of 80–90%, demonstrating high anticancer activity and tumor regression in nude mice.

Microneedles enable noninvasive and effective delivery of insulin through the skin. By penetrating the outer layer of the skin, microneedles achieve controlled release of insulin directly into the bloodstream or superficial tissues. This method not only enhances patient compliance but also offers a convenient alternative to traditional injections, potentially improving diabetes management and mimicking natural insulin secretion patterns more closely. Pere and colleagues used an SLA 3D printer to fabricate pyramid-shaped and conical microneedle patches, which were then coated with insulin using inkjet printing.^31^ In vitro insulin release studies showed rapid release, with 90–95% of insulin released within 30 min, making it suitable for insulin delivery. Liu et al. fabricated insulin-loaded microneedles via DLP printing, demonstrating in vitro and in vivo that the patches have a higher drug loading capacity, allowing for smaller patch sizes while still meeting the daily insulin requirements.^306^ These patches can effectively meet the needs for both postprandial and basal insulin by adopting different release strategies.

**Figure 6:**
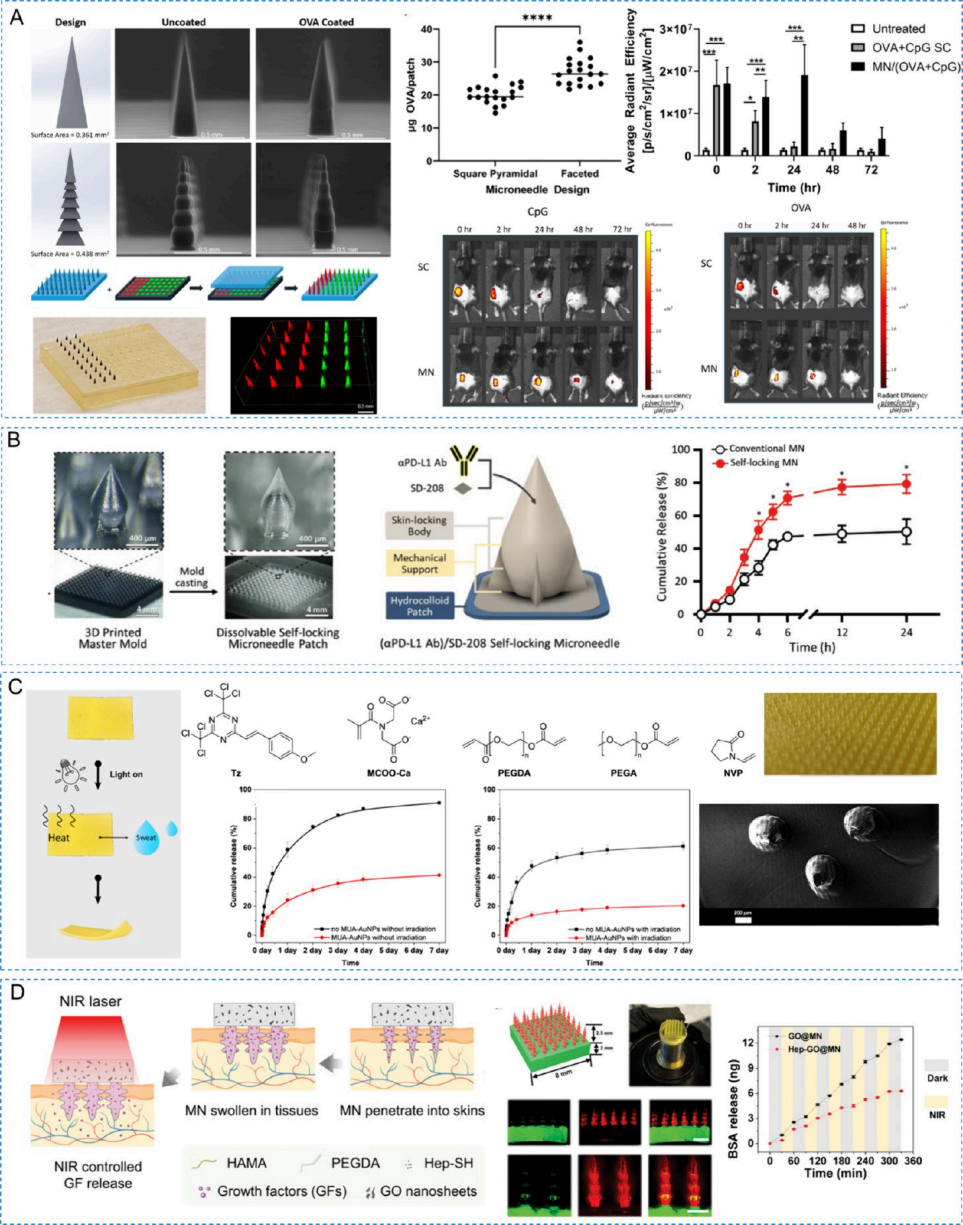
Photopolymerization 3D printing of customized microneedles for drug delivery. (A) 3D-Printed microneedles for transdermal vaccination elicit strong humoral and cellular immune responses. Reprinted or adapted with permission under a Creative Commons CC-BY from ref (90). Copyright 2021 PNAS. (B) Advanced self-locking microneedle patch for enhanced immunotherapy in melanoma. Reprinted or adapted with permission under a Creative Commons Attribution- NC-ND from ref (36). Copyright 2022 Advanced materials. (C) Advanced DLP 3D printed transdermal patches for enhanced drug delivery using gold nanoparticles and ion-induced SMP. Reprinted or adapted with permission under a Creative Commons CC-BY from ref (279). Copyright 2024 ACS Applied Materials & Interfaces. (D) Succulent-inspired microneedle patches for responsive drug delivery and enhanced biocompatibility using DLP 3D printing. Reprinted or adapted with permission from ref (280). Copyright 2024 Advanced Materials Technologies.

Through microneedle technology, vaccines and antibodies can be more effectively delivered to the superficial or deeper layers of the skin, thereby promoting immune response. This precise delivery method helps to enhance the stability and biological activity of vaccines and antibodies. As illustrated in Figure 6A, Caudill et al. used CLIP printing to design and manufacture a multifaceted microneedle array and coating mask devices, utilizing a resin composed of PEG350DMA with 2.5 wt % TPO as the PI.^90^ Compared to smooth square pyramid designs, the multifaceted microneedle design increases the surface area enhancing vaccine components (ovalbumin and CpG) retention in the skin and improving immune cell activation in lymph nodes.

Joo et al. utilized DLP-based 3D printing technology to fabricate a novel self-locking microneedle patch (Figure 6B).^36^ These patches were composed of PEGDA as the base material and methacrylated hyaluronic acid for the water-sensitive tips, featuring reversible shrink-swell characteristics. The self-locking microneedles were designed with a sharp skin-penetrating tip, a wide skin interlocking body, and a narrow base, with a flexible hydrocolloid patch to enhance skin penetration accuracy on irregular surfaces. These microneedle patches delivered a combination therapy of SD-208 and anti-PD-L1 antibodies, enhancing immunotherapy efficacy against melanoma. These self-locking microneedles successfully achieved precise skin insertion, adhesion, and microdose drug delivery functionality. Microneedles offer a noninvasive way for self-administered vaccines, making vaccination convenient and efficient worldwide, especially in resource-limited areas. This technology enhances vaccination safety and accessibility, leading to broader immunization coverage and better control of infectious disease spread.

Microneedles can be designed to release drugs in response to specific physiological conditions, disease states, or external stimuli, ensuring optimal drug delivery at the required time and location. Zhu and colleagues utilized DLP 3D printing technology to develop a novel prepolymer formulation (Figure 6C).^279^ This formulation includes PEGDA 700, 2-(4-methoxyphenyl)-4,6-bis(trichloromethyl)-1,3,5-triazine (Tz), gold nanoparticles, and ion-induced SMP, among other additives, for the fabrication of personalized transdermal patches. This method enables the precise manufacturing of microneedle arrays, which release heat under white light exposure, thereby promoting sweat production and enhancing drug delivery efficiency. The addition of Na^+^ helps regulate the curvature of the patch, while N-vinyl-2-pyrrolidinone optimizes its rheological properties. These advancements aim to improve the adhesion and conformity of the patches to curved skin surfaces, potentially enhancing therapeutic efficacy. Sun et al. utilized DLP technology to manufacture succulent-inspired microneedle patches (Figure 6D).^280^ The base material of the patches consisted of PEGDA, while the tips were composed of a combination of PEGDA and methacrylated hyaluronic acid, exhibiting reversible shrink-swell properties. This design facilitated effective penetration into the skin and long-term tissue adhesion. Integration of heparin sulfate enabled sustained release of growth factors, and graphene oxide allowed drug release under NIR control, demonstrating excellent biocompatibility and promoting proliferation, migration, and angiogenesis of human umbilical vein endothelial cells.

Lin et al. have developed a multimicrochannel microneedle microporation (4M) platform using surface PμSL for efficient and safe intracellular delivery and chemotherapy.^307^ This platform employs a cone-shaped microneedle array with through-microchannels, fabricated using high temperature low viscosity (HTL) yellow-5 resin. The microchannels facilitate the electrophoretic transport of charged molecules (such as cationic doxorubicin) into tumor cells at low voltages, enhancing drug delivery efficacy while reducing systemic toxicity. The 4 M platform significantly improves drug delivery efficiency and safety in both in vitro and in vivo experiments for solid tumor therapy.

Vat photopolymerization can be combined with microfluidic control to achieve 3D printed microneedles for customized drug delivery.^308^ Yeung et al. employed SLA and class IIa biocompatible resin (Formlabs) to manufacture hollow microneedle devices with integrated microfluidic functionalities for transdermal drug delivery. This approach enabled homogeneous mixing of multiple fluids and transdermal delivery of mixed solutions under varying flow rates. The printed devices exhibited high precision, consistency, and repeatability, as validated by scanning electron microscopy of multiple hollow microneedle array designs. Mechanical penetration and fracture tests confirmed the mechanical robustness of the microneedles for practical applications. The microfluidic-enabled microneedle device demonstrated uniform mixing of multiple fluids at different flow rates and transdermal delivery of mixed solutions. Comparative studies using colored dye solutions highlighted the adjustable control over the relative concentration of transported solutes. Ex vivo confocal laser scanning microscopy on porcine skin models further confirmed the ability to regulate and administer drugs through the skin of the platform.

Microneedles serve dual purposes as they can detect interstitial fluid and act as drug delivery devices, offering pathways for integrating noninvasive health monitoring and on-demand drug delivery in personalized medicine. Razzaghi et al. introduced a wearable therapeutic diagnostic device fabricated using DLP printing technology with PEGDA as the monomer, creating 3D-printed hollow microneedle arrays.^309^ These innovative MNs incorporate an integrated ultrasonic atomizer for precise on-demand drug delivery and feature biosensing capabilities for pH, glucose, and lactate detection. The summary of photopolymerization 3D printed customized microneedles, including those mentioned earlier, is presented in Table 2.

**Table 2:** Summary of Photopolymerization 3D Printed Customized Microneedles

Drug molecule	Drug delivery system	Materials	Drug loading mode	Drug release mode	3D Print Technology^ref^
**Indomethacin**	Microneedle	Trimethylene carbonate and ε-caprolactone	Immersion or direct filling	Immediate release	SLA^237^
**Insulin**	Microneedle	Dental SG (formlabs, usa)	Coating layers	Immediate release	SLA^31^
**Insulin**	Microneedle	Dental SG, (formlabs, usa)	Coating layers	Immediate release	SLA^258^
**Cisplatin**	Microneedle	Soluplus	Coating layers	Immediate release	SLA^32^
**Acetyl-hexapeptide 3**	Microneedle	PEGDA and vinylpyrrolidone	Suspend directly into the liquid resin	Immediate release	DLP^83^
**Ovalbumin and CpG**	Microneedle	PEGDMA	Coating layers	Sustained release	CLIP^90^
Dacarbazine	Microneedle	Poly(propylene fumarate) (ppf)	Direct dissolution	Modified release	PμSL^284^
Rhodamine B	Microneedle	PEGDA	Direct dissolution	Light-stimulated release	DLP^279^
Rhodamine B and VEGF	Microneedle	PEGDA	Direct dissolution or Immersion	Modified release and NIR stimulated release	DLP^280^
Anti-PD-L1 and SD-208	Microneedle	PEGDA	Direct dissolution	Modified release	DLP-based 3D printing^36^
Doxorubicin	Hallow microneedle	HTL yellow-5 resin	Direct filling	Electrical stimulated release	DLP-base 3D printing^307^
**Rhodamine B, fluorescein isothiocyanate and methylene blue**	Hallow microneedle device enabled with microfluidic chamber	Dental LT clear resin	Direct filling	Modified release	SLA^308^
**Verapamil hydrochloride**	Microneedle	Clear v4 resin (formlabs, usa)	Dip coating	Immediate release	SLA^310^
**Ceftriaxone sodium**	Microneedle	Biomed amber resin(formlabs, usa)	Direct filling	Immediate release	SLA^311^
**Silver and zinc oxide**	Microneedle	Acrylate-based polymer	Pulsed laser deposition	Sustained release	DLP^312^
**Diclofenac diethylamine**	Microneedle	3dm-castable resin	With commercial gels.	Modified release	DLP^313^
**Rhodamine B**	Microneedle	PEGDA	Immersion	Immediate release	PμSL^314^
**Fluorescently labeled nanoparticles**	Microneedle	IP-Q photoresist	Direct filling	Immediate release	DLP^315^
**Imiquimod**	Microneedle	PEGDA	Direct dissolution	Immediate release	DLP^316^
**Rhodamine B**	Microneedle	Hydroxybutyl methacrylated chitosan	Direct dissolution	Immediate release	DLP^317^

### 5.3 Drug-Eluting Implants and Medical Devices

Implantable drug delivery systems offer multiple advantages. They enable targeted drug release at the therapeutic target area, reducing systemic effects and enhancing treatment efficacy.^318^ Implants also allow for sustained, gradual drug release, minimizing drug concentration fluctuations compared to oral medications and maintaining stable drug concentrations in the treatment area.^319^ Based on PEGDA, Yang et al. utilized DLP printing to fabricate various implant shapes with sufficient drug-loading capacity and satisfactory biomechanical properties, demonstrating sustained release of sodium diclofenac over a 24-h period across different implant types.^29^ Ranganathan et al. used SLA printing to create femoral implants with composite resins containing varying fractions of PEG and PEGDA.^30^ Despite exposure to UV light, the implants retained the antibacterial properties of doxycycline, with the formulation containing 20% PEGDA and 80% PEG exhibiting optimal performance.

**Figure 7:**
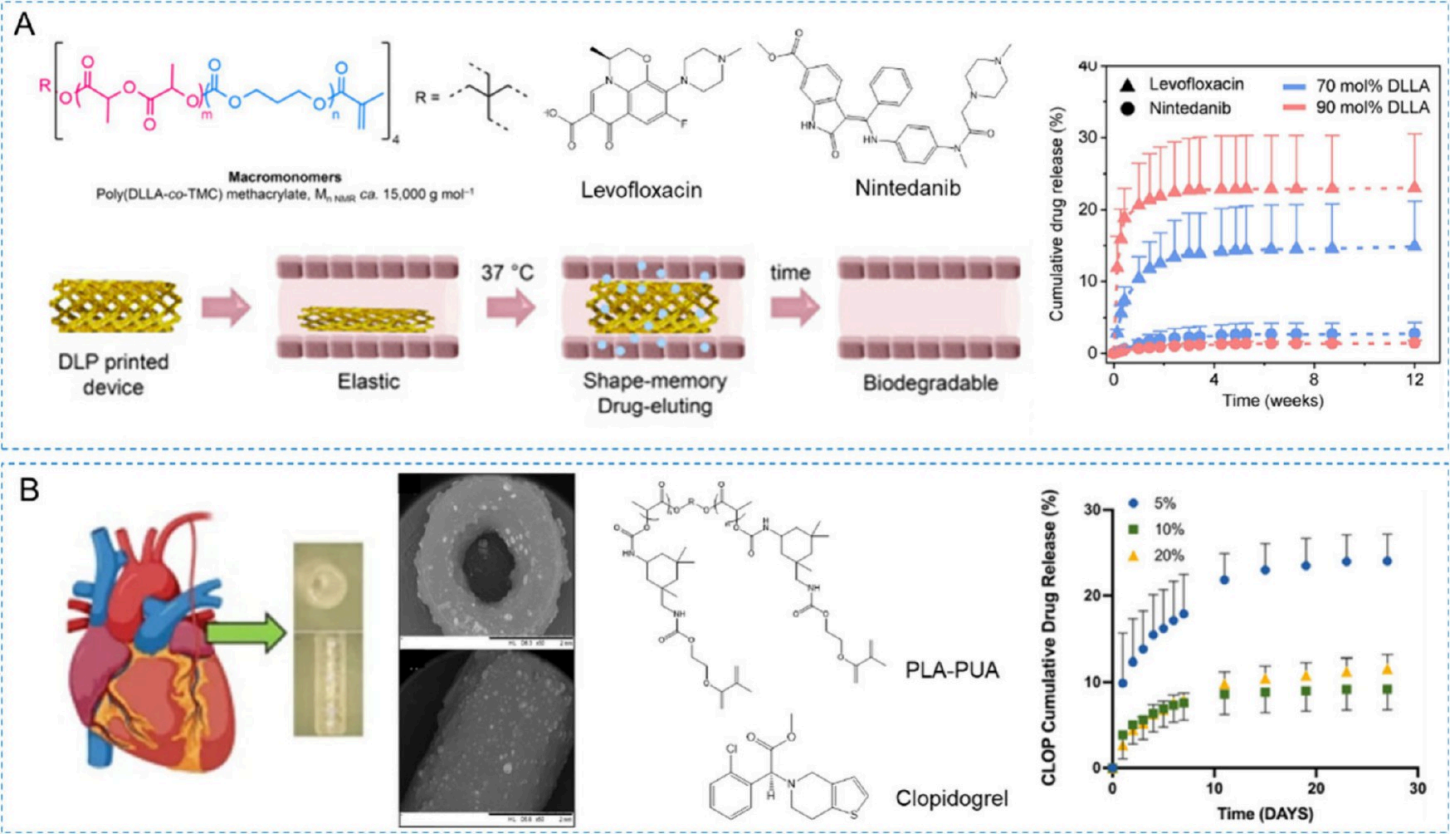
Photopolymerization 3D Printing of drug-eluting Implants and Medical Devices. (A) Development of biodegradable shape-memory elastomers for antibiotic delivery via DLP 3D printing. Reprinted or adapted with permission under a Creative Commons CC-BY from ref (33). Copyright 2023 Journal of controlled release. (B) Fabricating antithrombotic small-diameter cardiovascular grafts using DLP 3D printing. Reprinted or adapted with permission under a Creative Commons CC-BY from ref (34). Copyright 2024 Drug delivery and translational research.

As shown in Figure 7A, Paunovic et al. designed a biodegradable shape-memory elastomer by physically mixing poly(DLLA-*co*-TMC) with 70 or 90 mol % DLLA and 1 wt % levofloxacin, followed by fabrication using DLP printing.^33^ After incubation in PBS (pH 7.4) at 37 °C for one month, the scaffold exhibited sustained release characteristics, suggesting potential application in medical devices such as drug-eluting airway stents to administer antibiotics and prevent surgery-related infections. Triacca et al. utilized SLA to print a local drug delivery system for the using a flexible 80A resin (Formlabs) containing 0.5% w/v levofloxacin.^320^ In vitro experiments assessed the release profile of levofloxacin, the stability of the prototype implants, and their antimicrobial performance, showing that the drug diffused through the matrix prototype at a rate of 50% within 3 weeks.

Cardiovascular disease is a leading cause of global morbidity and mortality.^321^ Complications such as thrombosis with stent usage necessitate additional treatment or monitoring for some patients, highlighting the urgent need for new stent designs to improve patient outcomes. Photopolymerization-based 3D printing could potentially contribute to customization capabilities, higher precision for printing complex geometries, and improved material customization for biocompatibility. Lith et al. successfully utilized micro-CLIP printing technology to fabricate cardiovascular grafts.^207^ The printed bioresorbable scaffold retained the bioresorption and antioxidant properties of the resin material, methyl polydiolcitrate (mPDC), scavenging free radicals in vivo and demonstrating promising therapeutic effects. In their study, the scaffold was implanted into excised porcine arteries for in vivo experiments to evaluate its impact on enhancing arterial mechanical performance. The findings revealed that the 3D-printed scaffold displayed remarkable self-expansion capability and mechanical strength, effectively bolstering the mechanical properties of the arteries.

Adhami and colleagues utilized DLP 3D printing technology to fabricate small-diameter cardiovascular grafts containing the antithrombotic drug clopidogrel (CLOP) within a PLA–PUA(polyurethane acrylate)/L-PCL matrix (Figure 7B).^34^ Samples with the highest drug loading exhibited stable CLOP release over 27 days, with a minor burst release within the first 48 h, while reducing platelet deposition, demonstrating effective anticoagulation properties. Ding et al. utilized Micro-CLIP 3D printing technology to fabricate biodegradable vascular scaffolds (BVS) with fine struts measuring 62 μm.^285^ Using methacrylated poly(dodecanediol citrate) (mPDC) and methacrylated poly(octanediol citrate) (mPOC) materials, these scaffolds were successfully implanted into pig coronary arteries via a customized balloon catheter. The BVS demonstrated clinical performance comparable to the commercially available XIENCE drug-eluting stents within 28 days. Additionally, these scaffolds were coated with a biodegradable polymer (mPOC) that allowed for controlled release of the antirestenosis drug everolimus, achieving effective drug delivery and vascular regeneration while exhibiting excellent biocompatibility and antioxidant properties. Table 3 provides a summary of photopolymerization 3D printed drug-eluting implants and medical devices, including the ones discussed earlier.

**Table 3:** Summary of Photopolymerization 3D Printed Drug-Eluting Implants and Medical Devices

Drug molecule	Drug delivery device	Materials	Drug loading mode	Drug release mode	3D Print Technology^ref^
**Diclofenac sodium and ibuprofen**	Implant	PEGDA	Direct dissolution	Modified release	DLP^29^
**Doxycycline**	Implant	PEGDA, PEG	Direct dissolution	-	SLA^30^
**Levofloxacin and nintedanib**	Tracheal stent	Poly(DLLA-*co*-TMC)	Direct dissolution	Sustained release	DLP^33^
**Levofloxacin**	Ear implant	Flexible 80A Resin	Direct dissolution	Sustained release	SLA^312^
**Clop**	Cardiovascular graft	PLA–PUA/L-PCL	Direct dissolution	Sustained release	DLP^34^
**Dopamine**	Scaffold	Ormocomp	Dopamine releasing cell in alginate gel	Sustained release	Micro-SLA^322^
**Paclitaxel and Cisplatin**	Implant	HEMA, PEG-DMA	Direct dissolution	Sustained release	CLIP^239^
**Acetylsalicylic acid**	Scaffold	PEGDA	Direct dissolution	Immediate release	Micro-SLA^138^
**Lenti-BMP-2/GFP**	Scaffold	Methacrylated gelatin	Mixed into the gelatin solution	Sustained release	SLA^225^
**BMP-2 loaded PLGA microspheres**	Scaffold	PPF	Direct dissolution	Sustained release	Micro-SLA^187^
**Lanreotide**	Implant	Hydroxyethyl acrylate and PEGDA	Covalent connection	Modified release	DLP^250^
**Everolimus**	Scaffold	mPDC and mPOC	Coating layer	Sustained release	Micro-CLIP^285^

### 5.4 Drug Delivery Microrobots

**Figure 8:**
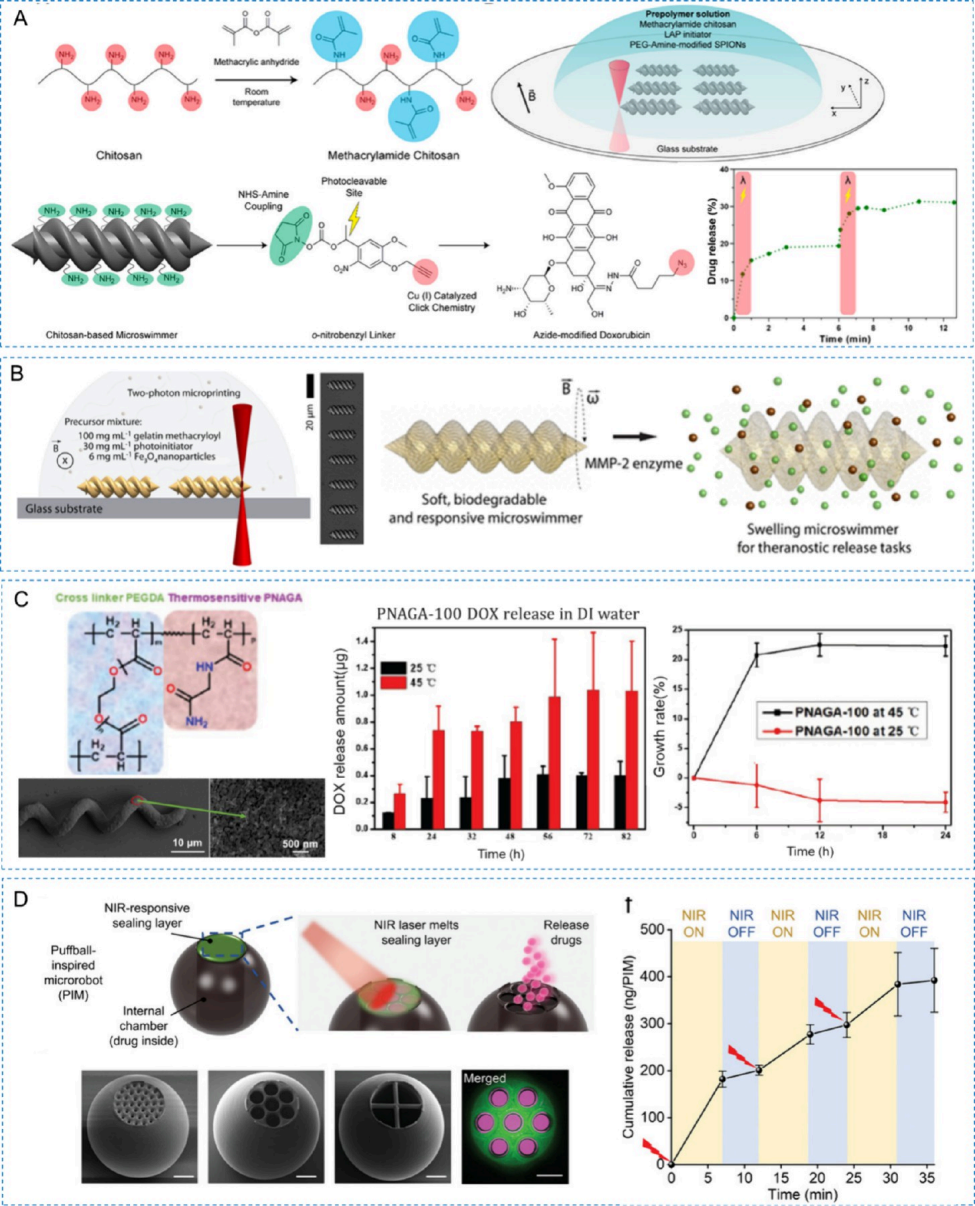
Photopolymerization 3D printing of microrobots for drug delivery. (A) Fabrication and functionalization of chitosan-based spiral microswimmers via 2PP for controlled drug delivery. Reprinted or adapted with permission under a Creative Commons CC-BY from ref (39). Copyright 2018 ACS Nano. (B) Development of biodegradable hydrogel-based microrobotic swimmers for responsive drug delivery. Reprinted or adapted with permission under a Creative Commons CC-BY from ref (40). Copyright 2019 ACS Nano. (C) Thermosensitive microrobots for controlled drug delivery and biomedical applications using 2PP 3D printing technology. Reprinted or adapted with permission under a Creative Commons CC-BY from ref (286). Copyright 2023 International journal of bioprinting. (D) PIMs for advanced drug delivery with precision targeting and on-demand release. Reprinted or adapted with permission under a Creative Commons CC-BY 4.0 from ref (238). Copyright 2022 Advanced materials.

The 2PP technology enables high-resolution 3D manufacturing at the micron level, allowing precise control over the shape, structure, and size of microdevices.^323^ This capability is particularly suitable for producing devices that require complex structures or specific functionalities. Compared to traditional free diffusion, microswimmers or microrobots can actively propel themselves, enhancing the transport efficiency of drugs to target tissues or cells, thereby improving therapeutic outcomes.^324^ Spiral microswimmers based on chitosan were fabricated using 2PP, enabling their 3D manufacturing at the microscale (Figure 8A).^39^ These microswimmers incorporate biocompatible superparamagnetic iron oxide nanoparticles, along with methylacrylamide chitosan as the photosensitive monomer and LAP as the photoinitiator, enabling multifunctional chemical moieties. Driven by a low-amplitude rotating magnetic field, the microswimmers exhibited an average speed of 3.34 ± 0.71 μm·s^–1^ with good controllability. Utilizing a light-triggered drug release mechanism, azide-modified doxorubicin was bound to the microswimmers, enabling light-triggered drug release. Microswimmers can integrate different functional modules, such as drug loading, targeted control, and pathological environment sensing, thereby enabling the combination or switching of various therapeutic strategies to enhance treatment flexibility and effectiveness.

In addition, biodegradable hydrogel-based microswimmers were developed to sense and respond to changes in the pathological microenvironment, such as the presence of the disease biomarker enzyme MMP-2, triggering accelerated therapeutic cargo release at tumor sites (Figure 8B).^40^ Beyond injectable microspheres, photopolymerization 3D printing is also used for fabricating drug infusion pumps and rapid reconstitution packages (RRPs). Gelatin methacryloyl was chosen as the photopolymer and LAP as the photoinitiator in devices produced via SLA, demonstrating superior performance in maintaining drug stability compared to glucagon stored in standard glass vials under identical temperature conditions. RRPs offer an alternative to manual reconstitution processes, specifically designed for medical emergency situations. Zhou et al. utilized 2PP 3D printing technology to fabricate thermosensitive microrobots based on poly-*N*-acryloyl glycinamide (PNAGA) hydrogels (Figure 8C).^286^ These microrobots, made with PNAGA-100 material, exhibited optimal swelling behavior at 45 °C, with a swelling rate of 22.5%. They were capable of controlled drug release, particularly of doxorubicin, with significantly higher release at 45 °C compared to 25 °C. Additionally, when decorated with Fe@ZIF-8 crystals, these biocompatible thermosensitive microrobots could swim under magnetic field control, demonstrating their potential in targeted drug delivery and biomedical applications.

As shown in Figure 8D, Song et al. employed IP-S photoresist and two-photon 3D microfabrication to design and manufacture puffball-inspired microrobots (PIMs).^238^ The PIMs feature a spherical main body for rolling propulsion and an internal chamber for drug loading. An automated dip-sealing method was used to apply a near-infrared responsive sealing layer on the top of the microrobots, which protects the drug payload and enables on-demand release. These capabilities allow PIMs to navigate precisely to target locations and achieve controlled drug delivery, demonstrating their potential and unique capabilities as targeted drug delivery systems. Table 4 summarizes photopolymerization 3D printed microrobots, including those mentioned earlier.

**Table 4:** Summary of Photopolymerization 3D Printed Microrobots and Other Unconventional Devices

Drug molecule	Drug delivery system	Materials	Drug loading mode	Drug release mode	3D Print Technology^ref^
**Doxycycline**	Microswimmer	Chitosan	Covalent connection	Light-stimulated release	2PP^39^
**Antierbb 2 antibody-tagged magnetic nanoparticles**	Microswimmer	Gelatin methacryloyl	Direct dissolution	Enzyme-stimulated release	2PP^40^
**Doxorubicin**	Microrobot	PNAGA	Direct dissolution	Temperature stimulated release	2PP^286^
**Rhodamine B**	Microrobot	Photoresist IP-S resin	Direct filling	Light-stimulated release	2PP^238^
**Lidocaine hydrochloride**	Bladder devices	Elastic resin from (formlabs, usa)	Direct filling or direct dissolution	Sustained release	SLA^50^
**Oleuropein**	Gels	PEGDMA	Direct dissolution	Sustained release	SLA^269^
**Polydiacetylene (PDA) nanoparticles**	Injectable particles	GELMA	Direct dissolution	Sustained release	DLP^325^
**Dexamethasone**	Punctal plugs	PEGDA, PEG	Direct dissolution	Sustained release	DLP^326^
**S-nitrosoglutathione (GSNO)**	Local NO delivery Hydrogel	PAA/F127/CNC	Immersion	Releases NO in a process triggered by water absorption	DLP^327^
**Dexamethasone-acetate and docetaxel**	Device	PEGDMA, PCLDMA	Direct dissolution	Sustained release	CLIP^240^
**Docetaxel and dexamethasone palmitate**	Brachytherapy spacer	Hydroxyethyl methacrylate and polyethylene glycol dimethacrylate	Direct dissolution	Sustained release	CLIP^88^
**Islatravir**	Implantable medical devices	Prototyping resin (UMA) and silicone resin (SIL 30)	Postfabrication absorption	Sustained release	CLIP^89^
-	Mucoadhesion device	HTM 140 M V2 3D printing photopolymer	Direct filling	Controlled orientation and intestinal retention	DLP^245^
**Salicylic acid**	Nose patches	PEGDA, PEG	Direct dissolution	Immediate release	SLA^328,329^

### 5.5 Unconventional Drug Delivery Devices

The application of photopolymerization 3D printing in the preparation of injection particles is an innovative method that allows the manufacturing of micrometer-scale particles by layer-by-layer stacking and UV curing of photosensitive material.^316^ This method allows for precise control over the size, shape, and structure of the particles, and enables the embedding of drugs, bioactive molecules, or other functional substances inside or on the surface of the particles.^317^ For example, Tao and colleagues designed a microgel functionalized with PDA nanoparticles and gelatin-methacryloyl (GelMA) hydrogel.^316^ This functional microgel was precisely manufactured using a 3D bioprinting process based on DLP, allowing for shape and size customization. PDA nanoparticles were mixed in the monomer solution and then 3D printed within the microgel. Pore-forming toxins (PFTs) could diffuse into the microgel and were subsequently captured and neutralized by the PDA nanoparticles. In a mouse model, local injection of the microgel facilitated tissue recovery after bacterial infection.

**Figure 9:**
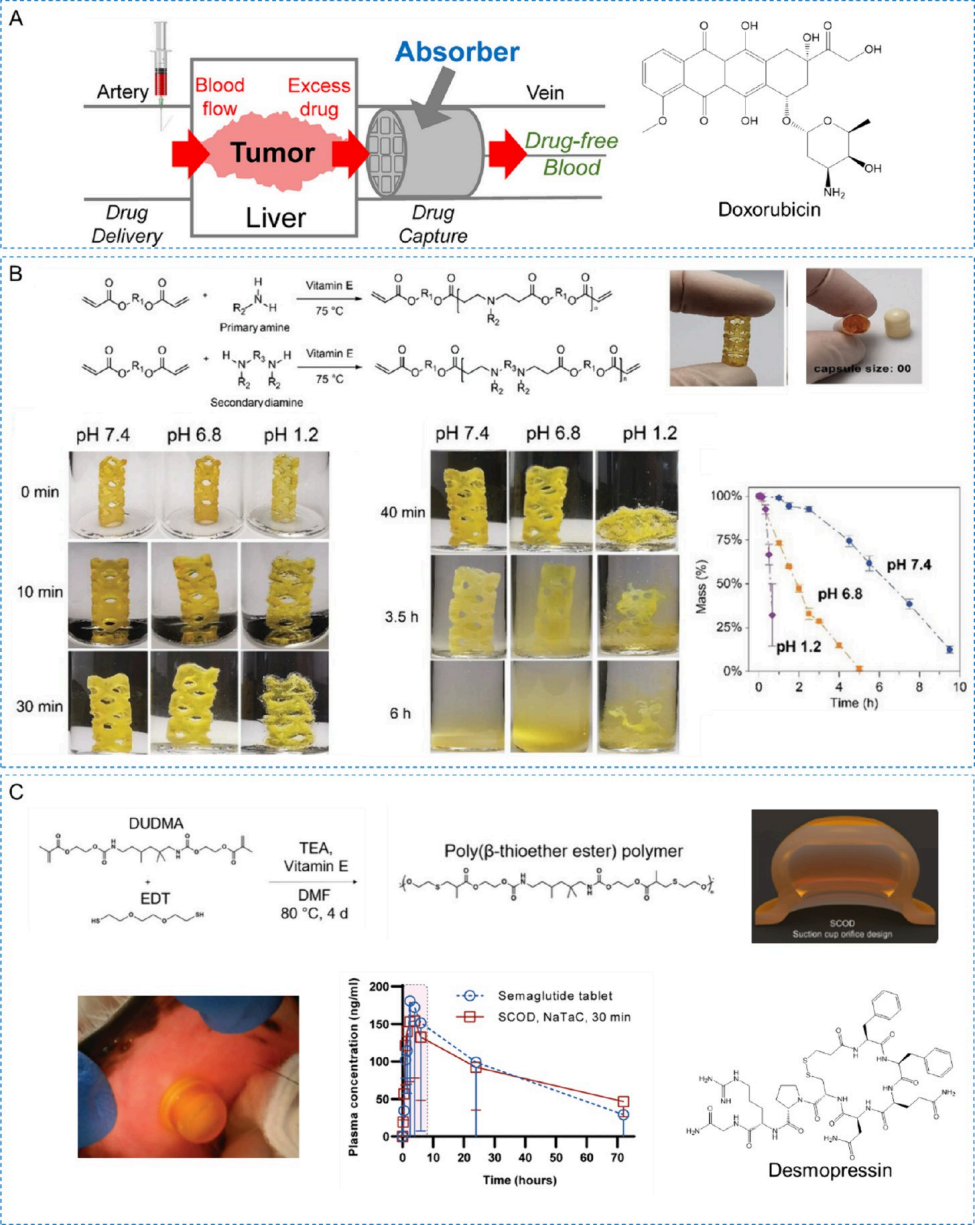
Photopolymerization 3D Printing for unconventional drug delivery applications. (A) Porous absorbers coated with nanostructured block copolymers for controlled drug capture. Reprinted or adapted with permission under a Creative Commons CC-BY from ref (38). Copyright 2019 ACS central science. (B) Fabrication of biodegradable elastomers for expandable oral drug delivery devices using digital light processing 3D printing. Reprinted or adapted with permission under a Creative Commons CC-BY 4.0 from ref (122). Copyright 2023 Advanced functional materials. (C) Development of DLP-printed buccal mucosal drug delivery patches enhanced with polyethylene for improved pharmacokinetics. Adapted with permission from ref (37). Copyright 2023 Science translational medicine.

In addition to the aforementioned drug delivery applications based on photopolymerization 3D printing, several published works primarily focused on other applications or unconventional devices also hold promise for drug delivery applications. Oh et al. utilized Clip technology and PEGDA as the monomer, with TPO as the photoinitiator, to construct and apply porous absorbers coated with nanostructured block copolymers (Figure 9A).^38^ Animal experiments verified that this device can successfully capture 64 ± 6% of the chemotherapy drug doxorubicin, thereby reducing systemic toxic side effects. The structure is coated with nanostructured block copolymers, with the outer blocks anchoring the polymer chains to the 3D printed support structure and the middle block having an affinity for the drug. The middle block is polystyrenesulfonate, which binds to doxorubicin to achieve drug capture. The structure and principles used in this experiment can also be applied as drug release devices, for instance, by preloading the stent coating with the drug and allowing it to be continuously released after vascular implantation.

We recently developed a series of photopolymerizable inks based on poly(β-aminoester) diacrylates and N-vinylpyrrolidone, which are DLP printed into biodegradable elastomers possessing good elasticity and high strength (Figure 9B).^122^ The printed scaffolds, measuring 2.2 cm in length and 0.8 cm in diameter, can be compressed and placed into size 00 capsules. The degradation performance of the elastomer was assessed under various pH conditions in animal models, showing potential for application in expandable oral drug delivery devices designed to degrade in the small intestine. Inspired by suckers structure of octopus, we used DLP printing to create buccal mucosal drug delivery patches in various shapes (Figure 9C).^37^ These patches can incorporate various excipients, including polyethylene (PE), to promote drug diffusion by disrupting the cellular tissue and lipid barrier of the mucosa. The synergistic effects between mechanical stretching and chemical action of PE enhance drug diffusion into highly vascularized tissues, facilitating entry into systemic circulation. Using desmopressin as a model drug, pharmacokinetic evaluations showed bioavailability of 3.2% and 4.1% after 10 and 30 min of application, respectively, which is 25 to 35 times higher than that of commercial oral medications. By precisely controlling material composition and printing processes, buccal mucosal drug delivery patches with biocompatibility and controllable degradation characteristics were obtained. This strategy holds the promise to improve efficiency and bioavailability for macromolecule drug delivery, while also offering new solutions for personalized therapy. Table 4 also includes a summary of photopolymerization 3D printed unconventional devices, including those referenced earlier.

## 6 Conclusions and Future Perspectives

Overall, significant advancements in the design of customized drug delivery systems have been achieved, owing to the rapid evolution of photopolymerization-based 3D printing for both techniques and corresponding biomaterials. The past decade has witnessed great enhancement of both printing resolution and printing speed for photopolymerization 3D printing, which are crucial for personalized drug delivery applications. For example, the invention of CLIP has revolutionized SLA/DLP, achieving printing rates up to 100 times faster than traditional methods.^84^ Innovations like heat-assisted DLP and iCLIP further allow the use of high-viscosity resins, broadening the range of printable formulations.^85^ Volumetric 3D printing methods such as CAL and xolography represent another leap forward, enabling rapid fabrication of complex three-dimensional structures without the need for layer-by-layer assembly or support materials.^106,110^ Particularly, xolography offers x-y resolution of 20 μm, achieving a voxel volume of 0.55 μm^3^, which is comparable with that of high-end SLA printers. Shifting from macro to micro scale, 2PP technology enables the creation of complex objects with nanosize suitable for smarter drug delivery.^98^ These technologies will, if not already, facilitate the development of drug delivery systems tailored to individual patient needs, although some of them have not yet been employed.

Without suitable biocompatible and biodegradable photopolymers, these advanced techniques wound not been put into force for drug delivery applications. Fortunately, numerous photopolymers have been developed specifically for photopolymerization 3D printing in past decade. These polymeric materials show tunable biodegradability, mechanical properties, and functionality, which make the application of advanced 3D printing techniques in drug delivery reality. For example, with good cytocompatibility, fast photopolymerization speed, and flexible architecture/functional groups, PEG-based photopolymers are widely explored for 3D printed drug formulations, scaffolds and devices· PEGDA-based 3D printed microneedles and scaffolds with controlled drug release capabilities have been developed for sustained drug delivery.^136,137^ The ring-opening copolymerization among different monomers like DLLA, CL and TMC and the resin formulation design greatly enhance the mechanical properties of the resulting 3D printed biodegradable elastomers, allowing the development of new drug-eluting medical devices.^155,157,174^ Recent advancements in controlled ROP synthesis of PPF with well-defined molecular weight and architectures, greatly enhanced its mechanical properties and flexibility in polymer design, facilitating functional scaffold fabrication.^184^ The step-growth polycondensation based polymers like PBAEs can be easily synthesized and functionalized for photopolymerization, which have shown great potential in protein delivery and gene therapy.^199−203^ While being biocompatible, these biodegradable photopolymers can be engineered to degrade at controlled rates for target drug release.

Based on parallel advancements of photopolymerization 3D printing techniques and the 3D printable biomaterials, we have observed new trends in the design of customized drug delivery systems:

i). In oral drug formulations, technologies like SLA and DLP have enabled the manufacturing of personalized tablets with controlled drug release profiles and multidrug capabilities, and most recently, volumetric printing has been employed for the tablet fabrication.^109^ Interestingly, the drug release kinetics can be affected by the 3D printing methods and the printed microstructures.^283^ By manipulating printing parameters, such as layer height, light density, and printing speed, it is possible to design drug delivery devices with customized release profiles, improving the efficacy of treatments.
ii). For transdermal drug delivery, microneedles fabricated via high-resolution photopolymerization techniques in particular CLIP offer noninvasive, efficient drug delivery with controlled release.^90^ Compared to traditional transdermal patches, microneedles can deliver high molecular weight drugs, including proteins and peptides. By adjusting the structure and materials of microneedles, controlled drug release can be achieved, which is particularly important for treatments requiring stable drug concentrations over time, such as insulin delivery for diabetes.^306^ Recently, 3D-printed microneedles show great potential in delivering various biopharmaceuticals, particularly vaccines and antibodies.^36,90^
iii). Implantable drug delivery systems benefit from improved mechanical properties and biodegradability of recently developed 3D printable polyester or polycarbonate-based photopolymers, allowing sustained and localized drug release.^318^ Implantable systems can deliver drugs directly to the diseased area, increasing the local concentration of the drug and reducing systemic side effects.^319^ They can also be used to control postoperative infections. By implanting antibacterial drug release systems at the surgical site, infections can be effectively prevented and treated, reducing the systemic use of antibiotics and lowering the risk of resistance.^33^ For cardiovascular diseases, drug eluting stents and heart valves can be fabricated, for example, to release antithrombotic drugs and reduce the risk of thrombosis.^207^
iv). The development of microrobots using 2PP technology has opened new avenues for targeted and stimuli-responsive drug delivery, combining the advantages of nanomedicine and photopolymerization 3D printing. These microrobots, designed as drug delivery systems, can respond to specific physical, chemical, or biological signals within the body to release drugs, such as under specific pH, temperature, or enzyme conditions, achieving targeted drug delivery.^39,40,286^ They demonstrate broad application prospects in the treatment of diseases like cancer.
v). Moreover, novel drug delivery devices such as smart capsules and buccal patches highlight the versatility of photopolymerization 3D printing in offering patient-specific treatment solutions with unconventional design. Smart capsules fabricated using photopolymerization 3D printing can dynamically adjust their size and shape to optimize drug release location within the digestive tract based on specific patient needs.^122^ Similarly, buccal patches manufactured with this technology feature intricate designs, enabling rapid drug release directly onto oral mucosa for localized treatment.^37^ These advancements enable more effective, personalized, and minimally invasive therapeutic approaches.

In summary, recent advancements in customized drug delivery systems based on photopolymerization 3D printing have brought significantly impact on the field of drug delivery and personalized medicine. The future of photopolymerization-based 3D printing in drug delivery looks promising, with potential applications extending beyond traditional pharmaceuticals into more complex and integrative therapeutic systems. Particularly, the integration of stimuli-responsive materials and nanotechnology within photopolymerization 3D printing systems opens new avenues for precision medicine, by realizing controlled and localized drug delivery while reducing dosage and mitigating systemic side effects.^330,331^ In addition, photopolymerization using advanced controlled reaction systems, such as reversible addition–fragmentation chain transfer (RAFT) polymerization,^332,333^ atom transfer radical polymerization (ATRP),^334^ and nitroxide-medicated polymerization (NMP)^335,336^ have attracted great attention for fabricating “living objects”. Owing to the accessibility to low-energy light sources (e,g., green or red light), these systems have shown promising applications in personalized drug delivery system.^337,338^ Together with the design of advanced photoinitiating systems,^339,340^ these controlled photopolymerization methods may broaden the applications of 3D-printed drug delivery systems.

Moreover, digital healthcare technologies, such as wearable sensors, can be combined with 3D-printed drug delivery devices to monitor physiological parameters in real-time, offering personalized and adaptive treatment.^341,342^ In addition, the combination of drug delivery systems with scaffolds for tissue engineering could accelerate the healing process and improve the quality of regenerated tissues.^343−345^ The design of novel liquid materials would be highly useful for the 3D printing of drug delivery systems by photopolymerization techniques, especially the most advanced ones.^156,346^ With the help of artificial intelligence, the design of 3D printed drug delivery systems could be more efficient and on target.^7,347^

While exhibiting considerable potential in the fabrication of drug delivery systems and devices, photopolymerization 3D printing also faces numerous challenges in the field. Note that the application of photopolymerization 3D printing in drug delivery systems remains predominantly at the research stage of in vitro test, with relatively few reports documenting its use for in vivo studies, indicating a significant gap before clinical research can be initiated. Unlike FDM and other 3D printing technologies that can directly utilize medical-grade polymers, photopolymerization 3D printing typically employs customized synthetic photopolymers. Ensuring the biocompatibility of these materials is paramount, particularly for drug delivery systems intended for long-term implantation. Because incompatible materials may trigger inflammation and allergic reactions, the toxicity of degradation products can affect the health of surrounding tissues. Consistency, stability, and cost control during the production process are critical factors for achieving clinical application. Although photopolymerization-based 3D printing can design specific drug release mechanisms, precise control over drug release rates still faces numerous challenges, influenced by the chemical properties of the drug and the structure of the materials. This requirement substantially increases the complexity of clinical trials and regulatory approval for photopolymerization 3D-printed drug delivery systems.

Additionally, a critical consideration is the trade-off between optimizing material properties (e.g., biodegradability, mechanical strength or elasticity) and adapting 3D printing methodologies to achieve superior print quality (e.g., reducing viscosity and printing time, or maintaining fidelity). While photopolymerization 3D printing is advantageous for small-scale production, issues related to cost and efficiency persist for large-scale manufacturing. Addressing these challenges will necessitate interdisciplinary collaboration, technological innovation, and supportive regulatory frameworks to facilitate the widespread adoption and advancement of photopolymerization 3D printing technology in the medical field. Overall, it is believed that the continuous research and innovation in this field will eventually lead to clinic breakthroughs in drug delivery systems that transform personalized medicine in reality.
